# Screening of the candidate genes related to low-temperature tolerance of *Fenneropenaeus chinensis* based on high-throughput transcriptome sequencing

**DOI:** 10.1371/journal.pone.0211182

**Published:** 2019-04-08

**Authors:** Xianhong Meng, Lijun Dong, Xiaoli Shi, Xupeng Li, Juan Sui, Kun Luo, Sheng Luan, Baolong Chen, Baoxiang Cao, Jiawang Cao, Jie Kong

**Affiliations:** 1 Laboratory for Marine Fisheries Science and Food Production Processes, Qingdao National Laboratory for Marine Science and Technology, Qingdao, China; 2 Key Laboratory of Sustainable Development of Marine Fisheries, Ministry of Agriculture, Yellow Sea Fisheries Research Institute, Chinese Academy of Fishery Sciences, Qingdao, China; 3 Shanghai Ocean University, Shanghai, China; Xiamen University, CHINA

## Abstract

In order to screen the candidate genes of *Fenneropenaeus chinensis* related to low-temperature tolerance, this research takes juvenile prawns of *F*. *chinensis* (P_40_) in low temperature stress group (4°C) and normal temperature group (18°C) as experimental materials. The results showed that a total of 127,939 Unigenes with average length of 1,190 bp were obtained by assembly, of which 46% were annotated in the Nr database. A total of 1,698 differentially expressed genes were screened by differential gene expression analysis, of which 920 genes showed up-regulated expression and 778 genes showed down-regulated expression. Both GO and KEGG enrichment analysis revealed that differentially expressed genes were enriched in spliceosomes, ribosomes, bile secretion, ABC transport pathways, and cellular nitrogen compound synthesis. A further in-depth analysis obtained 8 genes that may be associated with low-temperature traits of *F*. *chinensis*. Five of them displayed up-regulated expression, including *ATP-binding cassette protein C*, *acid ceramidase*, *glutathione transferase*, *C-type lectin* and *heat shock protein HSP70*. The remaining three genes, *γ-butyl betaine hydroxylase*, *β-hexosaminidase A* and *long chain fatty acid-CoA ligase* displayed down-regulated expression. Eight differentially expressed genes were randomly selected and the real time RT-PCR verification showed that their expression levels were consistent with the sequencing results, demonstrating the accuracy of the sequencing results. The results of this study provide basic data for revealing the molecular mechanisms of *F*. *chinensis* in response to low temperature stress and the molecular assisted breeding of *F*. *chinensis* in low temperature.

## Introduction

As one of the most important Chinese native mariculture species, Chinese fleshy shrimp (*Fenneropenaeus chinensis*) belongs to *Fenneropenaeus*, Penaeidae, Decapoda and Crustacea. It’s naturally distributed in the Yellow Sea, Bohai Sea (including the North West coast of Korea Peninsula), as well as Rongqiu Archipelago and Zhoushan Archipelago in the northern part of the East China Sea. [1]. As early as the 1950s, China conducted research on the reproduction and development of *F*. *chinensis* and carried out large-scale research on breeding and cultivation techniques for obtaining practical production techniques of this shrimp. In the late 1990s, China began to conduct the selective breeding. New variety of *F*. *chinensis* named ‘Huanghai No. 1’, ‘Huanghai No. 2’ and ‘Huanghai No. 3’ were cultivated [2–4].

The low-temperature tolerance traits have complex molecular regulation mechanisms because they are controlled by minorgene [5]. In recent years, with the rapid development of genomics and bioinformatics as well as other disciplines, high-throughput sequencing technology has developed more and more maturely, and it is widely used in the research of functional genes digging, molecular markers and signal transduction of aquatic animals. Huang et al. [6] used RNA-seq technology to analyze the transcriptome sequences of *Litopenaeus vannamei* in low temperature (13°C) and normal temperature (28°C) and obtained 72 differentially expressed genes. They discovered that serine/threonine kinases signaling pathways may play an important role in cold adaptation. Hu et al. [7] screened cold tolerance related genes of *Paralichthys olivaceus* using transcriptome sequencing and cloned CIPR and HMGB1.

As a cold-water shrimp, Chinese fleshy shrimps (*F*. *chinensis*) can live in the temperatures between 18°C to 30°C. The optimum temperature is 25°C and the extreme survival temperature for the shrimp is 4°C [8]. Meanwhile, the nursery temperature for *F*. *chinensis* shall be not less than 14°C. Therefore, the low temperature limits the breeding season and geographical area of *F*. *chinensis* and affect the production and efficiency of cultivation. In order to explore the molecular mechanisms of *F*. *chinensis* low temperature tolerance, differentially expressed genes under low temperature stress and low-temperature-related metabolic pathways were screened in the present study by comparing transcriptome information between 40-day prawns in normal temperature group (18°C) and low temperature groups (4°C). The results can provide theoretical basis for cultivation of new variety of *F*. *chinensis* with low-temperature tolerance.

## Materials and methods

### Experimental materials

The experiment was conducted in the Marine Genetics and Breeding Center of the Ministry of Agriculture, Yellow Sea Fisheries Research Institute, Chinese Academy of Fishery Sciences (Aoshanwei town, Jimo City, Qingdao) from April 2017 to June 2017. A total of 138 families of 40-day juvenile prawns belongs to G_12_ of new *F*. *chinensis* variety-‘Huanghai No. 2’ that produced in 2017 were selected as the experimental subjects with average body weight 0.150 ± 0.088g.

### Low temperature stress experiment

The experimental material was treated with low temperature stress and sampled by a temperature-adjustable refrigerator. Thirty individual shrimps were randomly selected from each family, and they were set up as the low temperature stress group (L) and same number of individuals of each family were selected for the normal temperature control group (N). The shrimps were cultivated in a 26.5 cm×20 cm×16.5 cm storage box. The water temperature in the low temperature stress group (L) firstly decreased from room temperature of 18°C to 14°C, and then decreased at a rate of 2°C per day until it dropped to 4°C and maintained at 4°C ± 0.5°C. The water temperature in the normal temperature control group (N) was maintained at 18 ± 1°C. During the cooling period, all shrimp family members were observed every two hours. The dead shrimps were collected and the time of death, family information, body length, and body weight were recorded. In the low-temperature stress group (L), water temperature was maintained at 4°C ± 0.5°C until 16 hours later. Three shrimps were randomly sampled from each L and N group and stored in sample protector for RNA (Takara, Japan). The cumulative survival time (CDH) of shrimp was calculated using ASReml-R (unpublished data). Considering the CDH and shrimp number alive at last, the FCQAS17G120264Y family with good performance of low-temperature tolerance traits were selected as target family. Three individuals in group L and group N of this family were used for transcriptome sequencing analysis.

### RNA extraction and transcriptome sequencing

Total RNA of each shrimp was extracted by the general Trizol method. RNA was examined for its mass and concentration by 1% agarose gel electrophoresis and Nanodrop ND-1000 spectrophotometer (Thermo, USA). The total RNA concentration shall be greater than 250 ng/μL. OD_260_/OD_280_ is between 1.8 and 2.2, and OD_260_/OD_230_ value shall be greater than or equal to 2.0, ensuring that RNA is not degraded and polluted. Finally, Total RNA was sent to Beijing Novogene company for Illumina HiSeq 2500 sequencing.

### Sequencing data assembly and annotation

After the quality analysis, the sequences with linker, low quality, and a ratio of N (where N indicates that the base information could not be determined) that was greater than 10% were removed from the raw data of the sequencing, thus clean data was obtained. By using Trinity software, non-parametric transcriptome splicing was performed using clean data. Each gene took the longest sequence obtained by splicing as Unigene, i.e. the reference sequence [9]. Then the Unigene sequences were compared and annotated with seven databases respectively, namely NCBI Nt, NCBI Nr, Pfam (http://pfam.sanger.ac.uk/), KOG (http://www.ncbi.nlm.nih.gov/CO/), Swiss-prot (http://www.ebi.ac.uk/uniprot/), KEGG (http://www.genome.jp/kegg/), and GO (http://www.geneontolorg.org/).

### Differential expression analysis and GO/KEGG enrichment analysis

FPKM (expected number of Fragments Per Kilobase of transcript sequence per Millions base pairs sequenced) refers to the number of fragments per Kilobase length from a gene in per million fragments [10]. In this study, the expression of Unigene was calculated by FPKM method. The unigenes using Trinity were used as the reference assembly for subsequent analysis (ref). The clean reads of each sample were mapped to ref. After comparing and collection of the results, the number of read counts for each sample mapped to each gene was obtained and the FPKM conversion was performed. According to the FPKM value, the edgR package in R language was used to screen the genes that were significantly differently expressed. The screening values were fold change>2 and *P*-value<0.05 [11]. Then GO and KEGG enrichment analysis (false discovery rate, FDR≤0.05) for differentially expressed genes were conducted by GOseq R and KOBAS software packages [12,13].

### Verification of transcriptome data by real time RT-PCR

Eight differentially expressed genes were randomly selected and specific primers were designed using Primer 5.0 software (Table 1). These genes were submitted to Sangon Biotech (Shanghai) Co, Ltd. for synthesis. By adopting TaKaRa relative fluorescence quantification kit, real time RT-PCR confirmatory experiment set up 3 parallel groups for each sample with the 18S rRNA as the reference gene. The dissolution curve was drawn after the reaction to ensure the specificity and accuracy of amplification. By using of 2^-△△Ct^ method the relative expression of genes were analyzed [14], and compared with the sequencing data.

**Table 1:** Genes and specific primers used for validation of RNA-seq data by real time RT-PCR.

Gene ID	Sequence(5'-3')
**C-70339**	ATGCTTGGTCTGCCCCC
	CGACAGTCTTGAGCCTTCCTT
**C-50465**	CCATCACCAACGACAAGGG
	TCCAGAGTGTTCTTCAGGCTCA
**C-55918**	CCGCTGAAGAGCACTGGAT
	CGCTTTTTCGGCTGACTG
**C-40114**	CCTCATTGACTTGTTCTCGGTA
	TTTCCTCTGGAGTGGTGGC
**C-40552**	GAGGGCATCTTTGAGCACAT
	GCATCATCTCGGTAAGGGTAGT
**C-60630**	ACTCCGTCGCCGAGAGAC
	CGAGGCTGGTGGTTGTTG
**C-22506**	CGACTCATTATGGGACGGATTA
	CTTTGCCCTCGTGCTGG
**C-31994**	AGACACGCCTTCGTTATGGA
	TGTAGACTTCGGGGACCTCA
**18S rRNA**	GGGGAGGTAGTGACGAAAAAT
	TATACGCTAGTGGAGCTGGAA

## Results

### Raw data assembly of sequencing

By the Illumina Hiseq 2500 sequencing for the total 6 samples in the low temperature group and the normal temperature group, a total of 267,810,398 original sequences were obtained, among which there were 257,507,504 clean reads and base Q30 (the value of Phred>30) accounted for 91%. After Trinity splicing, a total of 247,911 transcripts were obtained, with a splicing length ranged from 200 bp to 15,096 bp. There were 21,471 pri-miRNA with the average length of 759 bp. The N50 was 1,404 bp. Meanwhile, a total of 127,939 unigenes was obtained with splicing length ranged from 200 bp to 15,096 bp. There were 21,438 unigenes with length that was greater than 2,000 bp. The average length of unigenes were 1,190 bp, and the N50 was 1,826 bp (Table 2).

**Table 2:** Assembly statistic of transcripts and unigenes.

	Min Length	Mean Length/bp	Max Length/bp	N50^a^	N90^a^	Total
**Transcripts**	201	759	15,056	1,404	275	247,911
**Genes**	201	1190	15,056	1,826	532	127,939

^**a**^N50/N90 was sorted by the length of the spliced transcripts from long to short, and the length of the transcripts was accumulated to a length of no less than 50%/90% of the total spliced transcripts.

### Annotation of transcriptome data

In order to obtain comprehensive gene function information, we performed gene function annotations (Table 3) on the transcriptome data of *F*. *chinensis* by seven databases including Nr, Nt, Pfam, KOG/COG, Swiss-prot, KEGG and GO. The results showed that 58,938 unigenes out of a total of 127,939 unigenes had annotations in the Nr database, accounting for 46.06%. A total of 10,129 unigenes were noted in the above seven databases, accounting for 7.91%. A total of 83,086 unigenes were annotated at least one database, accounting for 64.94% of the total unigenes.

**Table 3:** Transcriptome data in seven databases in the annotate success rate statistics.

	Number of Genes	Percentage (%)
**Nr**	58,938	46.06
**Nt**	32,904	25.71
**KEGG**	32,108	25.09
**Swiss-prot**	50,232	39.26
**Pfam**	62,335	48.72
**GO**	63,360	49.52
**KOG**	28,091	21.95
**Seven Databases**	10,129	7.91
**At least one Database**	83,086	64.94
**Total unigenes**	127,939	100

### Screening of differentially expressed genes

**Fig 1:**
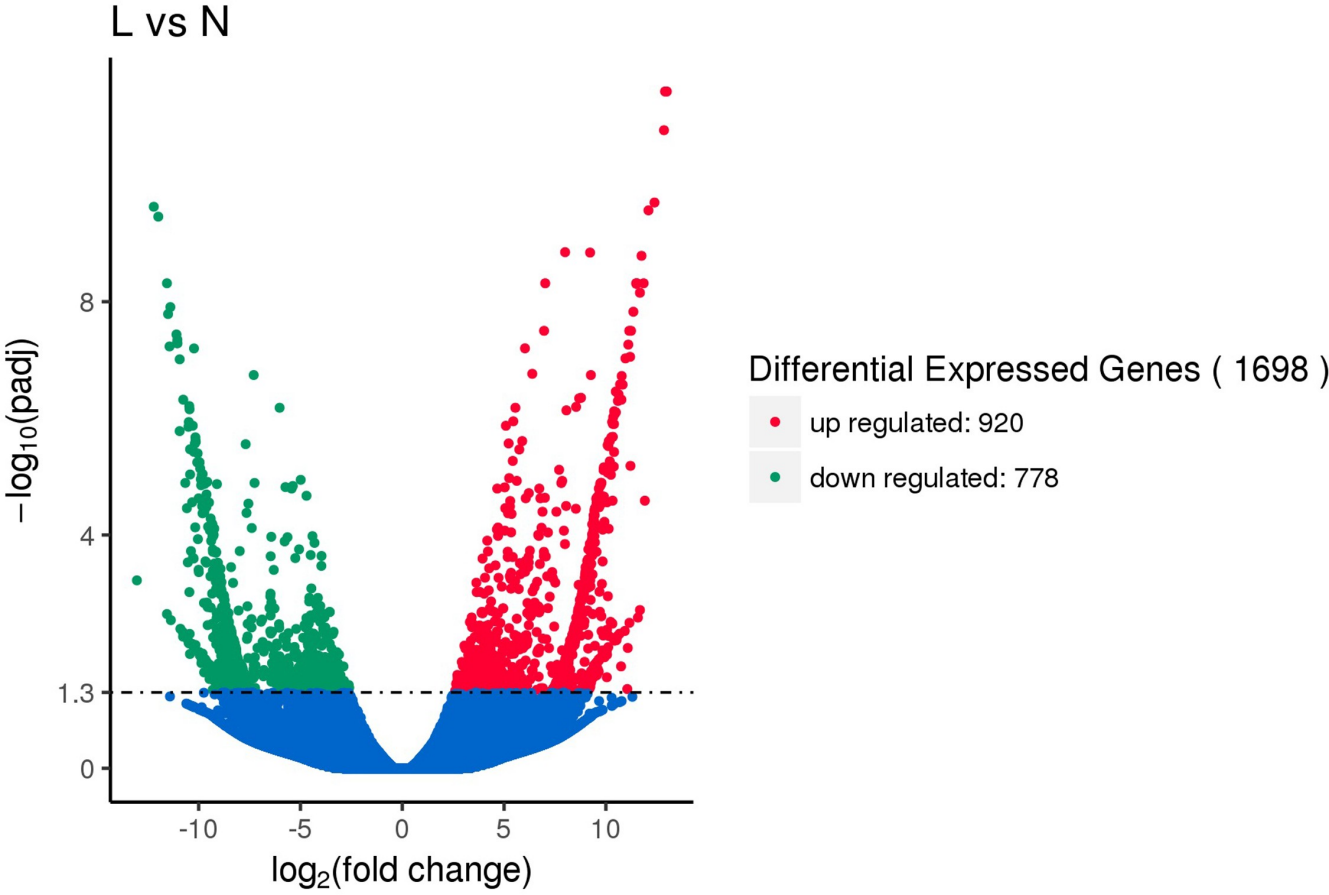
The ‘volcano plot’ of differentially expressed genes. The red dots represent genes with up-regulated expression. The green dots represent genes with down-regulated expression. The blue dots represent non differentially expressed genes.

In this study, edgeR was used to compare the differences in gene expression abundances between the low temperature group (4°C) and the normal temperature group (18°C), and then the differentially expressed gene volcano plots were drawn. As shown in Fig 1, a total of 1,698 diferentially expressed genes were screened, of which 920 genes showed up-regulated expression under low temperature stress and 778 genes showed down-regulated expression under low temperature stress. A total of 67% of the differentially expressed genes were annotated in the Nr database.

### Analysis of the most enriched GO terms

**Fig 2:**
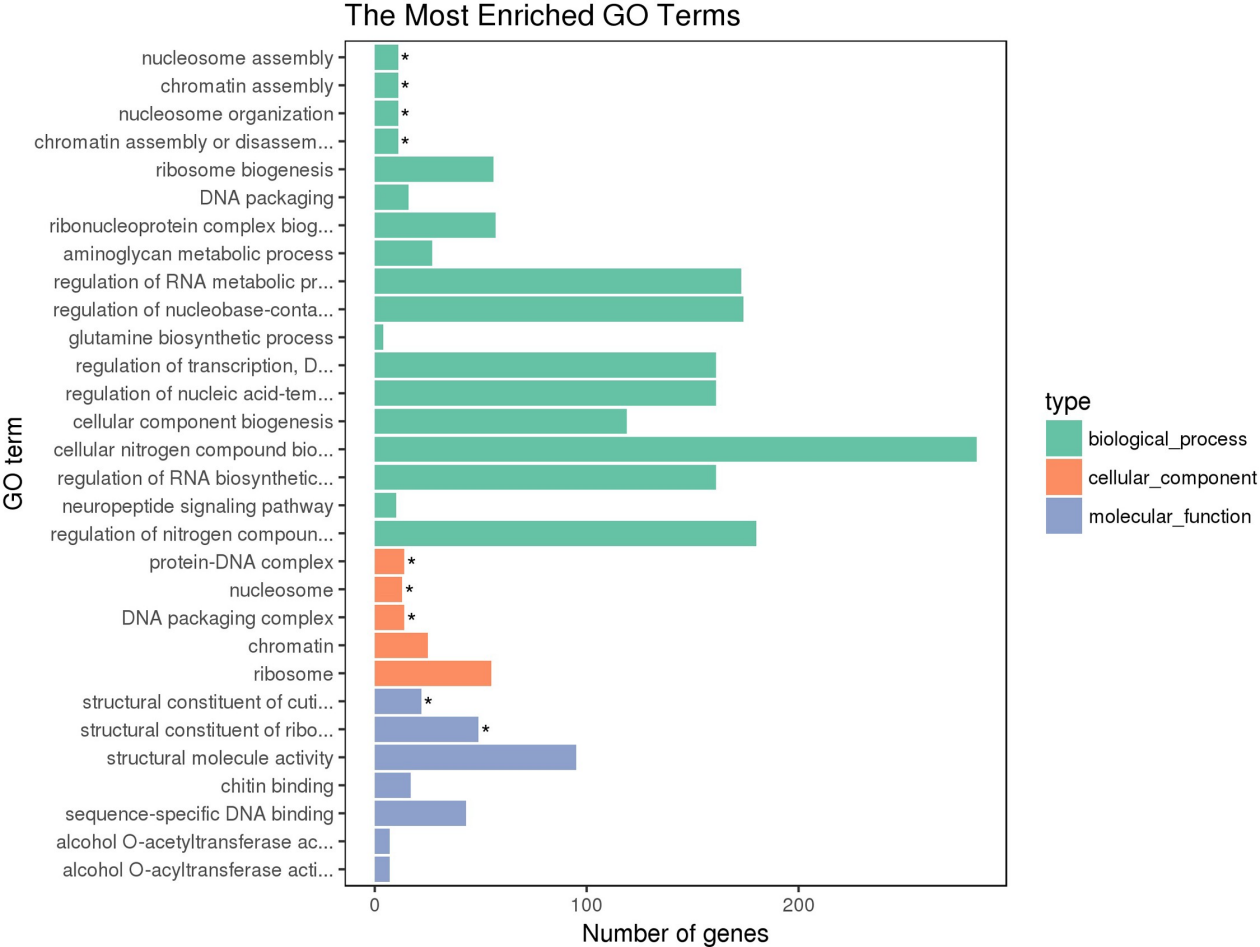
GO enrichment results of differentially expressed genes under cold challenge.

GOseq method was used in the study of this paper to enrich the 1,698 screened differentially expressed genes into 30 GO terms, and to count the number of genes in each GO term that were significantly enriched. The results are displayed as a histogram as shown in Fig 2. The differentially expressed genes are distributed in biological processes, cell classifications, and molecular functions, mainly in biological processes. In the biological processes, the three most abundantly differentially expressed genes are cellular nitrogen compound biosynthetic process, regulation of nitrogen compound metabolic process and regulation of nucleobase-containing compound metabolic process. In the cell classification, most of them are ribosomes. In molecular functions, the structural molecule activity contains the most differentially expressed genes.

### Enrichment analysis of KEGG differentially expressed genes

**Fig 3:**
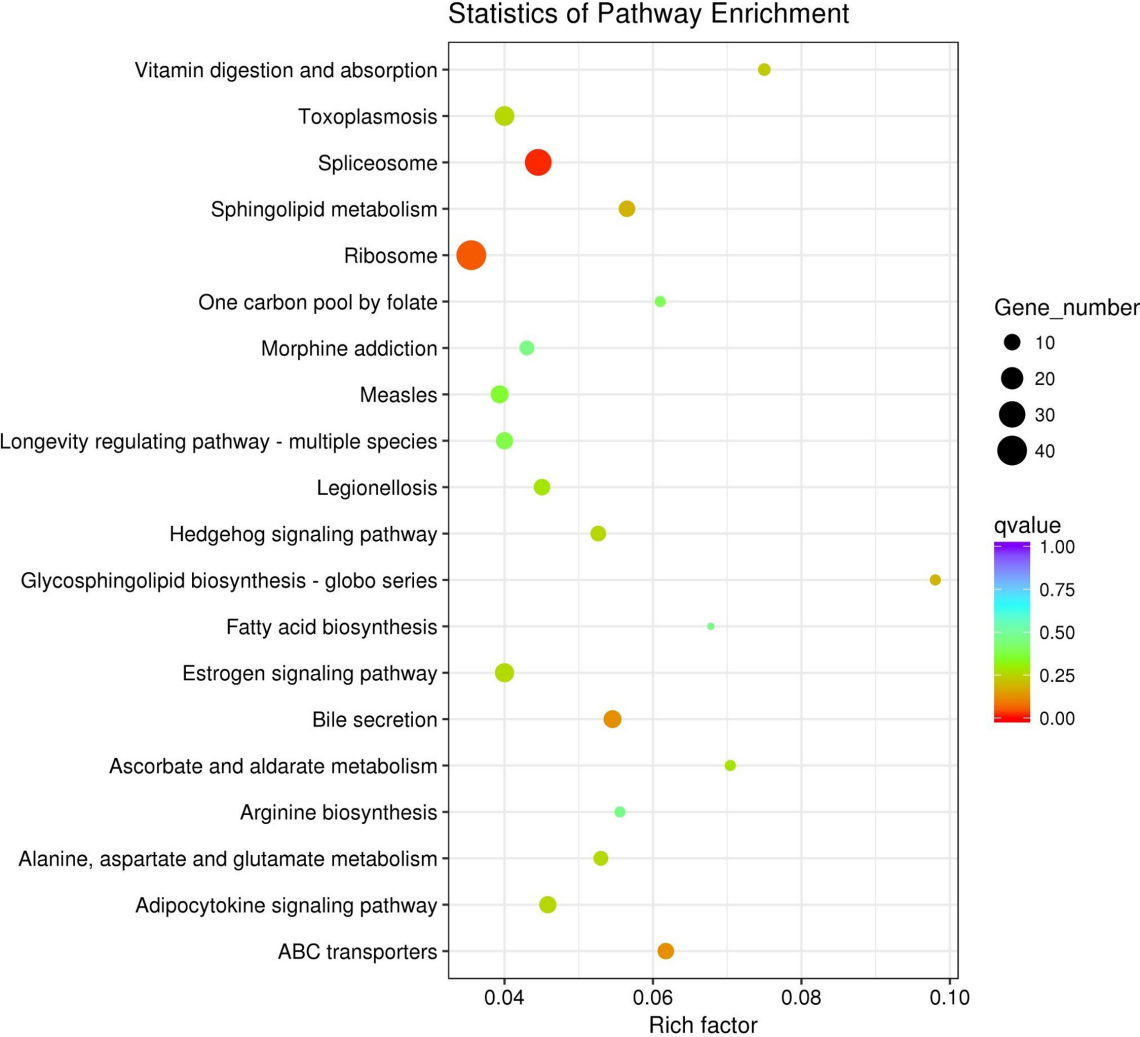
KEGG enrichment results of differentially expressed genes under cold challenge. The vertical axis indicates the name of pathway, and the horizontal axis indicates the corresponding Rich factor of the pathway. The size of q-value is represented by the color of dots. The smaller the q-value, the closer the color is to red. The number of differentially expressed genes contained in each pathway is represented by the size of dots.

In *vivo*, different genes coordinate to exercise their biological functions. Through the significant enrichment of metabolic Pathway can determine the major biochemical metabolic pathways and signal transduction pathways involved in differentially expressed genes [13]. KEGG is the main public database of Pathway. It compares the differentially expressed genes with the KEGG database, and selects the most significant top 20 enrichment pathways for scatter plots (Fig 3). Differentially expressed genes are abundant in Spliceosomes, Ribosomes, Bile Secretion, and ABC transporters.

### Candidate genes under low temperature

The study comprehensively analyzed the gene's differential expression fold (the higher the differential expression fold, the more significant the difference is) and the significant four pathways for differentially expressed gene enrichment, including ABC transport, sphingolipid metabolism, and glycosphingolipid biosynthetic pathway-Global Series, as well as genes involved in cold and environmental stress regulation reported in the References [15–28]. In the end, eight genes related to low temperature tolerance of Chinese fleshy shrimp were screened out from 1,698 differentially expressed genes, of which 5 showed up-regulated expression and 3 showed down-regulated expression (Table 4).

**Table 4:** Candidate genes in low temperature.

Gene ID	Gene Name	log_2_FoldChange	Regulation
**C-55918**	*AATP binding cassette protein C*, *ABCC3*	10.76253558	up
**C-66341**	*Acid ceramidase*, *ASA*H	10.59060336	up
**C-39485**	*Glutathione S- transferase*, *GST*	10.05836163	up
**C-49823**	*C-type lectin*	11.85426777	up
**C-50465**	*Heat shock protein HSP70*	6.384243125	up
**C-22506**	hypothetical protein: *γ-Butyl Betaine Dioxygenase*	-11.03745747	down
**C-58687**	*β-Hexosaminidase A*, *HEXA*	-6.023347561	down
**C-67709**	*Long chain fatty acid-CoA ligase*, *ACSBG2*	-11.55664899	down

### Verification of transcriptome data by real time RT-PCR

**Fig 4:**
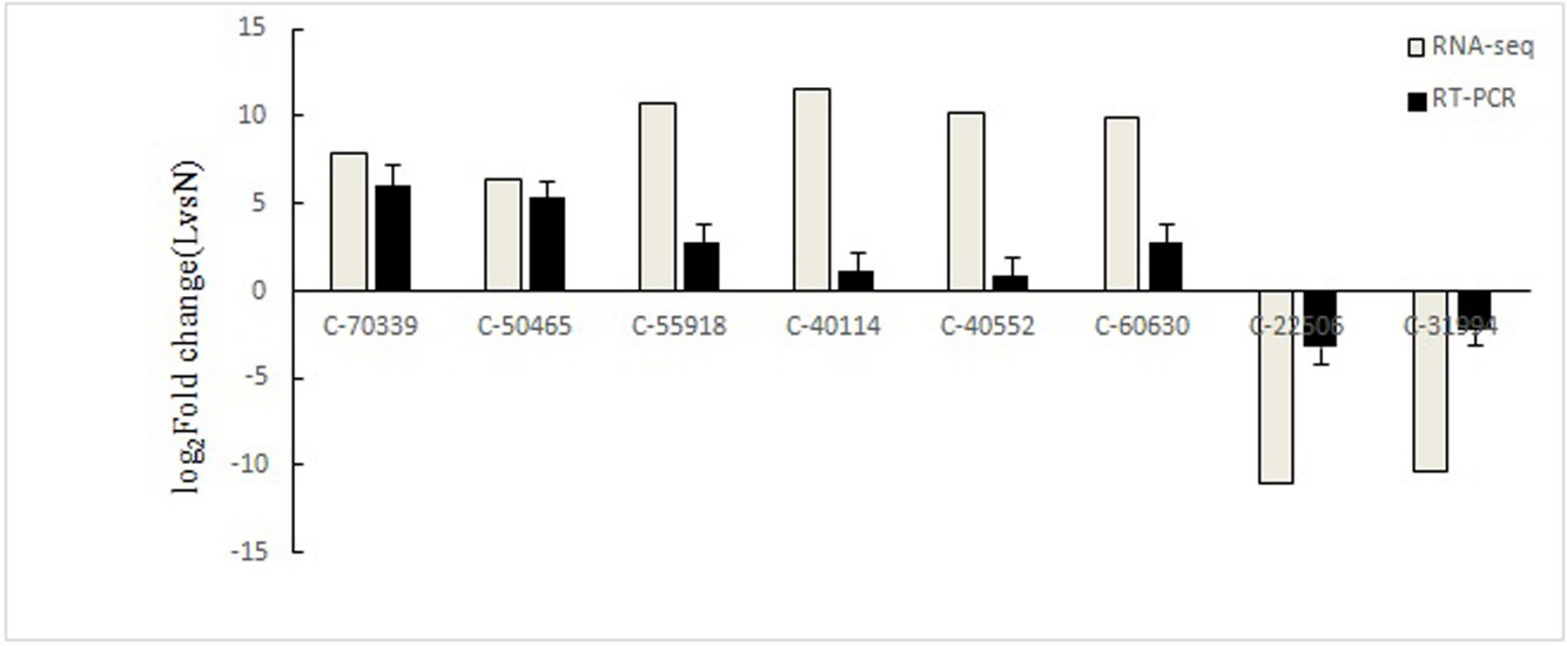
Verification of differentially expressed genes using real time RT-PCR.

Eight different genes were randomly selected, including three low-temperature candidate genes of *ATP-binding cassette protein C*, hypothetical protein: *γ-butyrobetaine dioxygenase*, and *heat shock protein HSP70*. Specific primers were designed for the experiment. The differentially expressed genes screened based on transcriptome data were verified by real time RT-PCR method. As shown in Fig 4, C-70339, C-50465, C-55918, C-40114, C-40552, and C-60630 all display up-regulated expression in real time RT-PCR and RNA-seq, while C-22506 and C-31994 show down-regulated expression in real time RT-PCR and RNA-seq, which also proves that the transcriptome data used in this study are reliable.

## Discussion

### Analysis on transcriptome of *F*. *chinensis* of low-temperature stress

It was reported that Illumina Hiseq 2500 was used in researches successfully such as small RNA, targeted sequencing, exons, transcriptome, metagenome, expression profile, genome sequencing, and long fragment sequencing and this technique is applicable to any species in the study of the group [15–16, 29–30]. In order to screen more genes that are specifically expressed under low temperature stress, the Chinese fleshy shrimp family with good low temperature tolerance was selected in this study according to the cumulative survival time (CDH). CDH can not only distinguish the different death temperatures, but also measure death at different time points at the same temperature [31–33], so it can accurately indicate the low-temperature traits.

Using Illumina Hiseq 2500 for high-throughput sequencing analysis of six individual shrimps with good tolerance to low temperature traits that belongs to the Chinese juvenile prawn family FCQAS17G120264Y, a total of 127,939 unigenes were obtained, of which 46% of the unigenes were annotated in the Nr database. A total of 1698 differentially expressed genes were obtained by differential gene expression analysis, of which 67% of the differentially expressed genes were annotated in the Nr database, which greatly enriched the transcriptome data pool of the Chinese fleshy shrimp. The functional annotations showed that differentially expressed genes were enriched in spliceosome, ribosome, bile secretion and ABC transport pathways, and the synthesis of cellular nitrogen compounds under low temperature stress. The current study have shown that temperature can affect the digestive system of *F*. *chinensis*, *L*. *vannamei*, *Scophthalmus maximus*, and *Epinephelussp* [17–20], while the ABC Transporters participate in the absorption, accumulation, and excretion of various toxic substances and play an important role in the immune defense process [21]. It is speculated that low temperature stress will cause differential expression of some genes in Chinese fleshy shrimp, and affect the digestive system, immune system and synthesis of nitrogen compounds such as fatty acids, amino acids and carbohydrates in cells.

### Candidate genes of *F*. *chinensis* in low temperature

Antifreeze protein (AFP)/Antifreeze glycoprotein (AFGP) found in fish is an early discovery of genes associated with low temperature tolerance traits [22]. There is still no report on the studies of related genes of juvenile prawn’s resistance to low temperatures currently. However, Peng Jinxia, Yin Qin et al. [23–27] studied and screened several genes related to low-temperature traits of *L*. *vannamei*, including *heat shock protein genes* (*HSPB1*, *HSP10*, *TCP-1-Beta*), *metallothioneins Gene* (*MT*), *coat protein gene* (*COPE*), *DEAD-box RNA helicase gene*, and *adenylyltransferase* (*ANT*2) [23–27]. Among them, *ANT*2 gene and *heat shock protein gene* have also been reported to be related to low temperature response in various aquatic animals such as *Scylla paramamosain*, *Dissostichus mawsoni*, and GIFT *Oreochromis niloticus*. [28, 34–35].

In this study, eight genes tolerance to low temperature Chinese fleshy shrimp were screened by analyzing the transcriptome information of the shrimps under low temperature stress. The *ATP-binding cassette protein* is encoded by the ABC gene and participates in the ABC transport mechanism in vivo. Fang Caiwang et al. found that the ABC transporter showed up-regulated expression when the scallop was exposed to cadmium stress, thus protecting the body from heavy metal toxicity [36]. It is consistent with the results of the study in this paper. *Glutathione transferase* (*GST*) can remove the excess free radicals (*ROS*) from the body and prevent oxidative damage. *GST* has been found to help plants resist environmental stresses such as hypothermia and is associated with resistance of *Venerupis philippinarum* to heavy metal stress [37–38]. The study in this paper found that *GST* expression increased under low temperature stress. It is speculated that low temperature induces a large number of *ROS* produced in the body, and high expression of *GST* can reduce the oxidative damage caused by low temperature. As an immune factor, *C-type lectin* plays an important role in innate immune defense [39]. Studies by Xia Dandan et al. showed that low or high temperature stress can cause changes in the expression of *C-type lectin* in *S*. *maximus* [40]. This study showed that *C-type lectin* was up-regulated under low temperature conditions. It was hypothesized that low temperature could damage the immune system of Chinese fleshy shrimp and cause increased expression of *C-type lectin*. *Gamma-butyrobetaine hydroxylase* is a key enzyme for the synthesis of L-carnitine in vivo. The main function of L-carnitine is to promote β-oxidation of long-chain fatty acids across the mitochondrial inner membrane and provide energy to cells. It has been researched that found L-carnitine is involved in cold stress responses in fish [41]. Heat shock proteins are a group of widely-existing heat stress proteins that are involved in the regulation of response to low temperature stress in shrimps of *L*. *vannamei* [23–24]. *β -hexosaminidase A* (*β-HEXA*) is a key enzyme in the Glycosaminoglycan Biosynthetic Pathway-Global Series. The phingoglycolipids have been reported to be involved in cell signal transduction and drought stress [42–43]. This study showed that *β-HEXA* is down-regulated under low temperature stress, suggesting that low temperature stress can hinder cell signal transduction. *Acid ceramidase* (*ASAH*) is involved in the regulation of the body's sphingolipid metabolism pathway, and it can be converted to sphingosine-1-phosphate hydrolysis of ceramide, and then regulate cell growth, aging, apoptosis and other functions. Studies have found that ASAH is associated with drought stress in plants [44]. The *long-chain fatty acid-CoA ligase* (*ACSBG2*) can regulate the biosynthesis of fatty acids and down-regulates them under low temperature stress. It is speculated that Chinese juvenile shrimp can increase the mobility of biofilm by reducing the synthesis of long chain fatty acids, thus responding to low temperature stress [45]. Therefore, these differentially expressed genes are closely related to low-temperature tolerance traits of Chinese juvenile shrimps. However, further verification is needed for more in-depth studies.

## Research meaning

Basing on transcriptome sequencing, this paper provided a comprehensive investigation of the differentially expressed genes of *F*. *chinensis* under low temperature stress. The research results are crucial for clarifying the molecular mechanism of low temperature tolerance of *F*. *chinensis* in obtaining the low temperature-resistance related genes. It will also provide theoretical support for low temperature molecularly assisted breeding of *F*. *chinensis* in China.

## Supporting information

S1 File Fc-low-tem-SNP-GO. (ZIP)

S2 File (XLS)

S3 File (XLS)
