# Supplementary material for: Screening of the candidate genes related to low-temperature tolerance of Fenneropenaeus chinensis based on high-throughput transcriptome sequencing
Source: PLoS One. 2019 Apr 8;14(4):e0211182. doi: 10.1371/journal.pone.0211182 (PMC6453463; doi:10.1371/journal.pone.0211182)
Supplement: S1 File — (ZIP) [file pone.0211182.s001.zip › Fc-low-tem-SNP-GO/DEG_KEGGenrichment/LvsN_down_kegg_web/LvsN_down.DEG_enriched_KEGG_pathway_API.html]

Pathway Enrichment

  

# The most enriched pathway terms

Statistic method: hypergeometric test

FDR correction method: Benjamini and Hochberg

| Term | Sample number | Background number | P-value | Corrected P-value | UniGenes | KO | Entrez ID | Ensembl ID | Gene name |
| --- | --- | --- | --- | --- | --- | --- | --- | --- | --- |
| One carbon pool by folate | 2 | 82 | 0.00160932782671 | 0.0659824408952 | Cluster-11054.62863 Cluster-11054.63374 | K00602 K00602 | NA | NA | purH purH |
| NF-kappa B signaling pathway | 2 | 160 | 0.00581492553478 | 0.119205973463 | Cluster-11054.46692 Cluster-11054.48110 | K03097 K03097 | NA | NA | CSNK2A CSNK2A |
| Ribosome biogenesis in eukaryotes | 2 | 265 | 0.0151139758074 | 0.201323474192 | Cluster-11054.46692 Cluster-11054.48110 | K03097 K03097 | NA | NA | CSNK2A CSNK2A |
| Measles | 2 | 305 | 0.0196413145553 | 0.201323474192 | Cluster-11054.46692 Cluster-11054.48110 | K03097 K03097 | NA | NA | CSNK2A CSNK2A |
| Fatty acid biosynthesis | 1 | 59 | 0.0410971632808 | 0.225853614062 | Cluster-11054.67709 | K15013 | NA | NA | ACSBG |
| Adherens junction | 2 | 467 | 0.0427365090919 | 0.225853614062 | Cluster-11054.46692 Cluster-11054.48110 | K03097 K03097 | NA | NA | CSNK2A CSNK2A |
| Herpes simplex infection | 2 | 501 | 0.0484386633442 | 0.225853614062 | Cluster-11054.46692 Cluster-11054.48110 | K03097 K03097 | NA | NA | CSNK2A CSNK2A |
| Fat digestion and absorption | 1 | 74 | 0.0511099120758 | 0.225853614062 | Cluster-11054.31994 | K13885 | NA | NA | SCARB1 |
| Ras signaling pathway | 2 | 525 | 0.0526215413273 | 0.225853614062 | Cluster-11054.48563 Cluster-11054.51840 | K17633 K05093 | NA | NA | RASAL2 FGFR2 |
| Vitamin digestion and absorption | 1 | 80 | 0.0550862473322 | 0.225853614062 | Cluster-11054.31994 | K13885 | NA | NA | SCARB1 |
| beta-Alanine metabolism | 1 | 95 | 0.0649557527515 | 0.233049617047 | Cluster-11054.51502 | K12259 | NA | NA | SMOX, PAO5 |
| Purine metabolism | 2 | 609 | 0.0682096440137 | 0.233049617047 | Cluster-11054.62863 Cluster-11054.63374 | K00602 K00602 | NA | NA | purH purH |
| Epstein-Barr virus infection | 2 | 668 | 0.0799565838928 | 0.252170764585 | Cluster-11054.46692 Cluster-11054.48110 | K03097 K03097 | NA | NA | CSNK2A CSNK2A |
| Ovarian steroidogenesis | 1 | 140 | 0.0939618073063 | 0.252312055657 | Cluster-11054.31994 | K13885 | NA | NA | SCARB1 |
| Fatty acid degradation | 1 | 147 | 0.0983938512857 | 0.252312055657 | Cluster-11054.67709 | K15013 | NA | NA | ACSBG |
| PPAR signaling pathway | 1 | 189 | 0.124543198261 | 0.252312055657 | Cluster-11054.67709 | K15013 | NA | NA | ACSBG |
| Arginine and proline metabolism | 1 | 189 | 0.124543198261 | 0.252312055657 | Cluster-11054.51502 | K12259 | NA | NA | SMOX, PAO5 |
| Prostate cancer | 1 | 205 | 0.134308023695 | 0.252312055657 | Cluster-11054.51840 | K05093 | NA | NA | FGFR2 |
| Aldosterone synthesis and secretion | 1 | 208 | 0.136127003439 | 0.252312055657 | Cluster-11054.31994 | K13885 | NA | NA | SCARB1 |
| Central carbon metabolism in cancer | 1 | 209 | 0.136732496929 | 0.252312055657 | Cluster-11054.51840 | K05093 | NA | NA | FGFR2 |
| Hepatitis C | 1 | 217 | 0.141561489552 | 0.252312055657 | Cluster-11054.31994 | K13885 | NA | NA | SCARB1 |
| Bile secretion | 1 | 220 | 0.143365525719 | 0.252312055657 | Cluster-11054.31994 | K13885 | NA | NA | SCARB1 |
| ErbB signaling pathway | 1 | 228 | 0.148158136941 | 0.252312055657 | Cluster-11054.23418 | K07365 | NA | NA | NCK |
| Adipocytokine signaling pathway | 1 | 240 | 0.15529780357 | 0.252312055657 | Cluster-11054.67709 | K15013 | NA | NA | ACSBG |
| Tight junction | 2 | 1010 | 0.157580469507 | 0.252312055657 | Cluster-11054.46692 Cluster-11054.48110 | K03097 K03097 | NA | NA | CSNK2A CSNK2A |
| Signaling pathways regulating pluripotency of stem cells | 1 | 264 | 0.169401578396 | 0.252312055657 | Cluster-11054.51840 | K05093 | NA | NA | FGFR2 |
| T cell receptor signaling pathway | 1 | 267 | 0.171148235307 | 0.252312055657 | Cluster-11054.23418 | K07365 | NA | NA | NCK |
| Lysine degradation | 1 | 269 | 0.172310672156 | 0.252312055657 | Cluster-11054.22506 | K00471 | NA | NA | E1.14.11.1 |
| Estrogen signaling pathway | 1 | 350 | 0.218069678078 | 0.308305406938 | Cluster-11054.74929 | K09571 | NA | NA | FKBP4\_5 |
| Insulin resistance | 1 | 374 | 0.231144986451 | 0.308562381056 | Cluster-11054.55436 | K15719 | NA | NA | NCOAT, MGEA5 |
| Axon guidance | 1 | 378 | 0.233303263726 | 0.308562381056 | Cluster-11054.23418 | K07365 | NA | NA | NCK |
| Pathogenic Escherichia coli infection | 1 | 506 | 0.299317475625 | 0.383500515644 | Cluster-11054.23418 | K07365 | NA | NA | NCK |
| Viral carcinogenesis | 1 | 626 | 0.356127240714 | 0.432426103974 | Cluster-11054.24956 | K16174 | NA | NA | MRPS18B, MRPS18-2 |
| MAPK signaling pathway | 1 | 635 | 0.360201079298 | 0.432426103974 | Cluster-11054.51840 | K05093 | NA | NA | FGFR2 |
| Spliceosome | 1 | 696 | 0.387154244376 | 0.432426103974 | Cluster-11054.27355 | K12897 | NA | NA | TRA2 |
| PI3K-Akt signaling pathway | 1 | 721 | 0.397876141754 | 0.432426103974 | Cluster-11054.51840 | K05093 | NA | NA | FGFR2 |
| Rap1 signaling pathway | 1 | 724 | 0.399150345346 | 0.432426103974 | Cluster-11054.51840 | K05093 | NA | NA | FGFR2 |
| Phagosome | 1 | 744 | 0.407577734015 | 0.432426103974 | Cluster-11054.31994 | K13885 | NA | NA | SCARB1 |
| Regulation of actin cytoskeleton | 1 | 753 | 0.411332147683 | 0.432426103974 | Cluster-11054.51840 | K05093 | NA | NA | FGFR2 |
| Endocytosis | 1 | 911 | 0.473567924495 | 0.485407122607 | Cluster-11054.51840 | K05093 | NA | NA | FGFR2 |
| Pathways in cancer | 1 | 1007 | 0.508181365756 | 0.508181365756 | Cluster-11054.51840 | K05093 | NA | NA | FGFR2 |
