# Supplementary material for: Screening of the candidate genes related to low-temperature tolerance of Fenneropenaeus chinensis based on high-throughput transcriptome sequencing
Source: PLoS One. 2019 Apr 8;14(4):e0211182. doi: 10.1371/journal.pone.0211182 (PMC6453463; doi:10.1371/journal.pone.0211182)
Supplement: S1 File — (ZIP) [file pone.0211182.s001.zip › Fc-low-tem-SNP-GO/DEG_KEGGenrichment/LvsN_kegg_web/LvsN.DEG_enriched_KEGG_pathway_API.html]

Pathway Enrichment

  

# The most enriched pathway terms

Statistic method: hypergeometric test

FDR correction method: Benjamini and Hochberg

| Term | Sample number | Background number | P-value | Corrected P-value | UniGenes | KO | Entrez ID | Ensembl ID | Gene name |
| --- | --- | --- | --- | --- | --- | --- | --- | --- | --- |
| Bile secretion | 6 | 220 | 8.00380021882e-05 | 0.00904429424726 | Cluster-11054.54514 Cluster-11054.54510 Cluster-11054.55919 Cluster-11054.55918 Cluster-11054.54276 Cluster-11054.31994 | K14158 K14158 K05666 K05667 K05667 K13885 | NA | NA | SLC5A1, SGLT1 SLC5A1, SGLT1 ABCC2 ABCC3 ABCC3 SCARB1 |
| One carbon pool by folate | 3 | 82 | 0.00238531270167 | 0.134770167644 | Cluster-11054.54624 Cluster-11054.62863 Cluster-11054.63374 | K00548 K00602 K00602 | NA | NA | metH, MTR purH purH |
| Mineral absorption | 3 | 113 | 0.00570751295987 | 0.214982988155 | Cluster-11054.54514 Cluster-11054.47412 Cluster-11054.54510 | K14158 K07213 K14158 | NA | NA | SLC5A1, SGLT1 ATOX1, ATX1, copZ, golB SLC5A1, SGLT1 |
| NF-kappa B signaling pathway | 3 | 160 | 0.0143591181891 | 0.2998255194 | Cluster-11054.46692 Cluster-11054.54104 Cluster-11054.48110 | K03097 K03097 K03097 | NA | NA | CSNK2A CSNK2A CSNK2A |
| ABC transporters | 3 | 162 | 0.0148311154612 | 0.2998255194 | Cluster-11054.55919 Cluster-11054.55918 Cluster-11054.54276 | K05666 K05667 K05667 | NA | NA | ABCC2 ABCC3 ABCC3 |
| Measles | 4 | 305 | 0.0159199390832 | 0.2998255194 | Cluster-11054.46692 Cluster-11054.54104 Cluster-11054.48110 Cluster-11054.50465 | K03097 K03097 K03097 K03283 | NA | NA | CSNK2A CSNK2A CSNK2A HSPA1\_8 |
| Epstein-Barr virus infection | 6 | 668 | 0.0189993310501 | 0.306703486952 | Cluster-11054.44996 Cluster-11054.50114 Cluster-11054.54104 Cluster-11054.48110 Cluster-11054.50465 Cluster-11054.46692 | K03036 K03029 K03097 K03097 K03283 K03097 | NA | NA | PSMD11, RPN6 PSMD4, RPN10 CSNK2A CSNK2A HSPA1\_8 CSNK2A |
| Insulin resistance | 4 | 374 | 0.0304267582552 | 0.351989344737 | Cluster-11054.55436 Cluster-11054.52075 Cluster-11054.44711 Cluster-11054.40313 | K15719 K15719 K16172 K16172 | NA | NA | NCOAT, MGEA5 NCOAT, MGEA5 IRS1 IRS1 |
| Toxoplasmosis | 4 | 375 | 0.0306794747665 | 0.351989344737 | Cluster-11054.44184 Cluster-11054.55722 Cluster-11054.40552 Cluster-11054.50465 | K05719 K05719 K00461 K03283 | NA | NA | ITGB1 ITGB1 ALOX5 HSPA1\_8 |
| Aldosterone-regulated sodium reabsorption | 2 | 92 | 0.034494867453 | 0.351989344737 | Cluster-11054.44711 Cluster-11054.40313 | K16172 K16172 | NA | NA | IRS1 IRS1 |
| Inositol phosphate metabolism | 3 | 232 | 0.0368776896898 | 0.351989344737 | Cluster-11054.40113 Cluster-11054.66262 Cluster-11054.40114 | K00911 K01099 K00911 | NA | NA | ITPK E3.1.3.36 ITPK |
| Adipocytokine signaling pathway | 3 | 240 | 0.0400769100369 | 0.351989344737 | Cluster-11054.67709 Cluster-11054.44711 Cluster-11054.40313 | K15013 K16172 K16172 | NA | NA | ACSBG IRS1 IRS1 |
| Carbohydrate digestion and absorption | 2 | 102 | 0.0414337018356 | 0.351989344737 | Cluster-11054.54514 Cluster-11054.54510 | K14158 K14158 | NA | NA | SLC5A1, SGLT1 SLC5A1, SGLT1 |
| Drug metabolism - cytochrome P450 | 2 | 105 | 0.0436092993479 | 0.351989344737 | Cluster-11054.39485 Cluster-11054.39487 | K00799 K00799 | NA | NA | GST, gst GST, gst |
| Ribosome biogenesis in eukaryotes | 3 | 265 | 0.0509431785741 | 0.355929061903 | Cluster-11054.46692 Cluster-11054.54104 Cluster-11054.48110 | K03097 K03097 K03097 | NA | NA | CSNK2A CSNK2A CSNK2A |
| Type II diabetes mellitus | 2 | 116 | 0.0519355447922 | 0.355929061903 | Cluster-11054.44711 Cluster-11054.40313 | K16172 K16172 | NA | NA | IRS1 IRS1 |
| Longevity regulating pathway - multiple species | 3 | 275 | 0.0556495576951 | 0.355929061903 | Cluster-11054.44711 Cluster-11054.40313 Cluster-11054.50465 | K16172 K16172 K03283 | NA | NA | IRS1 IRS1 HSPA1\_8 |
| Metabolism of xenobiotics by cytochrome P450 | 2 | 122 | 0.0566966647279 | 0.355929061903 | Cluster-11054.39485 Cluster-11054.39487 | K00799 K00799 | NA | NA | GST, gst GST, gst |
| Ovarian steroidogenesis | 2 | 140 | 0.0718273147824 | 0.371703241537 | Cluster-11054.40552 Cluster-11054.31994 | K00461 K13885 | NA | NA | ALOX5 SCARB1 |
| Herpes simplex infection | 4 | 501 | 0.0724742107107 | 0.371703241537 | Cluster-11054.46692 Cluster-11054.59082 Cluster-11054.48110 Cluster-11054.54104 | K03097 K12896 K03097 K03097 | NA | NA | CSNK2A SFRS7 CSNK2A CSNK2A |
| PI3K-Akt signaling pathway | 5 | 721 | 0.0759528776297 | 0.371703241537 | Cluster-11054.44184 Cluster-11054.55722 Cluster-11054.44711 Cluster-11054.40313 Cluster-11054.51840 | K05719 K05719 K16172 K16172 K05093 | NA | NA | ITGB1 ITGB1 IRS1 IRS1 FGFR2 |
| Proteasome | 2 | 145 | 0.0762385112313 | 0.371703241537 | Cluster-11054.44996 Cluster-11054.50114 | K03036 K03029 | NA | NA | PSMD11, RPN6 PSMD4, RPN10 |
| Chemical carcinogenesis | 2 | 147 | 0.0780265926671 | 0.371703241537 | Cluster-11054.39485 Cluster-11054.39487 | K00799 K00799 | NA | NA | GST, gst GST, gst |
| Leishmaniasis | 2 | 153 | 0.0834687643491 | 0.371703241537 | Cluster-11054.44184 Cluster-11054.55722 | K05719 K05719 | NA | NA | ITGB1 ITGB1 |
| Pertussis | 2 | 157 | 0.0871595620543 | 0.371703241537 | Cluster-11054.44184 Cluster-11054.55722 | K05719 K05719 | NA | NA | ITGB1 ITGB1 |
| Phosphatidylinositol signaling system | 3 | 334 | 0.0873276028846 | 0.371703241537 | Cluster-11054.40113 Cluster-11054.66262 Cluster-11054.40114 | K00911 K01099 K00911 | NA | NA | ITPK E3.1.3.36 ITPK |
| Longevity regulating pathway - worm | 3 | 341 | 0.0915006146119 | 0.371703241537 | Cluster-11054.54952 Cluster-11054.44711 Cluster-11054.40313 | K10798 K16172 K16172 | NA | NA | PARP IRS1 IRS1 |
| Arrhythmogenic right ventricular cardiomyopathy (ARVC) | 3 | 342 | 0.0921034580799 | 0.371703241537 | Cluster-11054.44184 Cluster-11054.55722 Cluster-11054.44073 | K05719 K05719 K04962 | NA | NA | ITGB1 ITGB1 RYR2 |
| Regulation of lipolysis in adipocytes | 2 | 177 | 0.106300740546 | 0.414206333852 | Cluster-11054.44711 Cluster-11054.40313 | K16172 K16172 | NA | NA | IRS1 IRS1 |
| Axon guidance | 3 | 378 | 0.114865045507 | 0.432658338077 | Cluster-11054.44184 Cluster-11054.55722 Cluster-11054.23418 | K05719 K05719 K07365 | NA | NA | ITGB1 ITGB1 NCK |
| Cell adhesion molecules (CAMs) | 2 | 192 | 0.121320948693 | 0.442234425881 | Cluster-11054.44184 Cluster-11054.55722 | K05719 K05719 | NA | NA | ITGB1 ITGB1 |
| Glutathione metabolism | 2 | 199 | 0.128499262207 | 0.453763019668 | Cluster-11054.39485 Cluster-11054.39487 | K00799 K00799 | NA | NA | GST, gst GST, gst |
| Glycosphingolipid biosynthesis - globo series | 1 | 51 | 0.148680426531 | 0.468545723823 | Cluster-11054.57109 | K00718 | NA | NA | FUT1\_2 |
| ECM-receptor interaction | 2 | 220 | 0.150585482747 | 0.468545723823 | Cluster-11054.44184 Cluster-11054.55722 | K05719 K05719 | NA | NA | ITGB1 ITGB1 |
| Selenocompound metabolism | 1 | 52 | 0.151312593213 | 0.468545723823 | Cluster-11054.54624 | K00548 | NA | NA | metH, MTR |
| Legionellosis | 2 | 222 | 0.152727249926 | 0.468545723823 | Cluster-11054.34236 Cluster-11054.50465 | K14416 K03283 | NA | NA | HBS1 HSPA1\_8 |
| mTOR signaling pathway | 2 | 224 | 0.154875013254 | 0.468545723823 | Cluster-11054.44711 Cluster-11054.40313 | K16172 K16172 | NA | NA | IRS1 IRS1 |
| Calcium signaling pathway | 3 | 439 | 0.157564048719 | 0.468545723823 | Cluster-11054.40113 Cluster-11054.44073 Cluster-11054.40114 | K00911 K04962 K00911 | NA | NA | ITPK RYR2 ITPK |
| Small cell lung cancer | 2 | 235 | 0.166788071034 | 0.474979854573 | Cluster-11054.44184 Cluster-11054.55722 | K05719 K05719 | NA | NA | ITGB1 ITGB1 |
| Fatty acid biosynthesis | 1 | 59 | 0.169512277747 | 0.474979854573 | Cluster-11054.67709 | K15013 | NA | NA | ACSBG |
| Spliceosome | 4 | 696 | 0.172337823341 | 0.474979854573 | Cluster-11054.59082 Cluster-11054.50465 Cluster-11054.27355 Cluster-11054.18182 | K12896 K03283 K12897 K12850 | NA | NA | SFRS7 HSPA1\_8 TRA2 PRPF38B |
| Adherens junction | 3 | 467 | 0.178589812623 | 0.480491638724 | Cluster-11054.46692 Cluster-11054.54104 Cluster-11054.48110 | K03097 K03097 K03097 | NA | NA | CSNK2A CSNK2A CSNK2A |
| Glycosphingolipid biosynthesis - lacto and neolacto series | 1 | 66 | 0.187323368966 | 0.492268388212 | Cluster-11054.57109 | K00718 | NA | NA | FUT1\_2 |
| RNA degradation | 2 | 262 | 0.19662594579 | 0.502580942749 | Cluster-11054.37559 Cluster-11054.40917 | K12604 K00962 | NA | NA | CNOT1, NOT1 pnp, PNPT1 |
| Non-alcoholic fatty liver disease (NAFLD) | 3 | 499 | 0.20348641414 | 0.502580942749 | Cluster-11054.37506 Cluster-11054.44711 Cluster-11054.40313 | K00237 K16172 K16172 | NA | NA | SDHD, SDH4 IRS1 IRS1 |
| Fat digestion and absorption | 1 | 74 | 0.207213662424 | 0.502580942749 | Cluster-11054.31994 | K13885 | NA | NA | SCARB1 |
| Pathogenic Escherichia coli infection | 3 | 506 | 0.209038091232 | 0.502580942749 | Cluster-11054.44184 Cluster-11054.55722 Cluster-11054.23418 | K05719 K05719 K07365 | NA | NA | ITGB1 ITGB1 NCK |
| Vitamin digestion and absorption | 1 | 80 | 0.221812640681 | 0.522183924936 | Cluster-11054.31994 | K13885 | NA | NA | SCARB1 |
| Hypertrophic cardiomyopathy (HCM) | 3 | 544 | 0.239713530544 | 0.552808754112 | Cluster-11054.44184 Cluster-11054.55722 Cluster-11054.44073 | K05719 K05719 K04962 | NA | NA | ITGB1 ITGB1 RYR2 |
| Glycosylphosphatidylinositol(GPI)-anchor biosynthesis | 1 | 91 | 0.247885765805 | 0.552942954586 | Cluster-11054.36172 | K05290 | NA | NA | PIGK |
| Dilated cardiomyopathy (DCM) | 3 | 556 | 0.249558324636 | 0.552942954586 | Cluster-11054.44184 Cluster-11054.55722 Cluster-11054.44073 | K05719 K05719 K04962 | NA | NA | ITGB1 ITGB1 RYR2 |
| beta-Alanine metabolism | 1 | 95 | 0.257149698211 | 0.556906195888 | Cluster-11054.51502 | K12259 | NA | NA | SMOX, PAO5 |
| Longevity regulating pathway | 2 | 319 | 0.261203790992 | 0.556906195888 | Cluster-11054.44711 Cluster-11054.40313 | K16172 K16172 | NA | NA | IRS1 IRS1 |
| Purine metabolism | 3 | 609 | 0.293642233648 | 0.6029598912 | Cluster-11054.63374 Cluster-11054.62863 Cluster-11054.40917 | K00602 K00602 K00962 | NA | NA | purH purH pnp, PNPT1 |
| Estrogen signaling pathway | 2 | 350 | 0.296538154685 | 0.6029598912 | Cluster-11054.74929 Cluster-11054.50465 | K09571 K03283 | NA | NA | FKBP4\_5 HSPA1\_8 |
| FoxO signaling pathway | 2 | 352 | 0.29881198148 | 0.6029598912 | Cluster-11054.44711 Cluster-11054.40313 | K16172 K16172 | NA | NA | IRS1 IRS1 |
| Neurotrophin signaling pathway | 2 | 358 | 0.305626270182 | 0.605890675974 | Cluster-11054.44711 Cluster-11054.40313 | K16172 K16172 | NA | NA | IRS1 IRS1 |
| Citrate cycle (TCA cycle) | 1 | 133 | 0.339682526357 | 0.661795266868 | Cluster-11054.37506 | K00237 | NA | NA | SDHD, SDH4 |
| AMPK signaling pathway | 2 | 402 | 0.355109490133 | 0.676823488909 | Cluster-11054.44711 Cluster-11054.40313 | K16172 K16172 | NA | NA | IRS1 IRS1 |
| Fatty acid degradation | 1 | 147 | 0.367731013914 | 0.676823488909 | Cluster-11054.67709 | K15013 | NA | NA | ACSBG |
| Arachidonic acid metabolism | 1 | 153 | 0.379385655595 | 0.676823488909 | Cluster-11054.40552 | K00461 | NA | NA | ALOX5 |
| Shigellosis | 2 | 425 | 0.380496015879 | 0.676823488909 | Cluster-11054.44184 Cluster-11054.55722 | K05719 K05719 | NA | NA | ITGB1 ITGB1 |
| Base excision repair | 1 | 156 | 0.385132518941 | 0.676823488909 | Cluster-11054.54952 | K10798 | NA | NA | PARP |
| Rap1 signaling pathway | 3 | 724 | 0.390068762313 | 0.676823488909 | Cluster-11054.44184 Cluster-11054.55722 Cluster-11054.51840 | K05719 K05719 K05093 | NA | NA | ITGB1 ITGB1 FGFR2 |
| Phagosome | 3 | 744 | 0.406617257839 | 0.676823488909 | Cluster-11054.44184 Cluster-11054.55722 Cluster-11054.31994 | K05719 K05719 K13885 | NA | NA | ITGB1 ITGB1 SCARB1 |
| Regulation of actin cytoskeleton | 3 | 753 | 0.414022041407 | 0.676823488909 | Cluster-11054.44184 Cluster-11054.55722 Cluster-11054.51840 | K05719 K05719 K05093 | NA | NA | ITGB1 ITGB1 FGFR2 |
| Insulin signaling pathway | 2 | 460 | 0.418285884244 | 0.676823488909 | Cluster-11054.44711 Cluster-11054.40313 | K16172 K16172 | NA | NA | IRS1 IRS1 |
| Sphingolipid metabolism | 1 | 177 | 0.42390397923 | 0.676823488909 | Cluster-11054.66341 | K12348 | NA | NA | ASAH1 |
| Cysteine and methionine metabolism | 1 | 187 | 0.441501287026 | 0.676823488909 | Cluster-11054.54624 | K00548 | NA | NA | metH, MTR |
| PPAR signaling pathway | 1 | 189 | 0.444955996629 | 0.676823488909 | Cluster-11054.67709 | K15013 | NA | NA | ACSBG |
| Arginine and proline metabolism | 1 | 189 | 0.444955996629 | 0.676823488909 | Cluster-11054.51502 | K12259 | NA | NA | SMOX, PAO5 |
| cGMP-PKG signaling pathway | 2 | 487 | 0.446631113388 | 0.676823488909 | Cluster-11054.44711 Cluster-11054.40313 | K16172 K16172 | NA | NA | IRS1 IRS1 |
| Bacterial invasion of epithelial cells | 2 | 496 | 0.45590833502 | 0.676823488909 | Cluster-11054.44184 Cluster-11054.55722 | K05719 K05719 | NA | NA | ITGB1 ITGB1 |
| MicroRNAs in cancer | 2 | 508 | 0.468138275988 | 0.676823488909 | Cluster-11054.44711 Cluster-11054.40313 | K16172 K16172 | NA | NA | IRS1 IRS1 |
| Prostate cancer | 1 | 205 | 0.471838600867 | 0.676823488909 | Cluster-11054.51840 | K05093 | NA | NA | FGFR2 |
| Aldosterone synthesis and secretion | 1 | 208 | 0.476732829612 | 0.676823488909 | Cluster-11054.31994 | K13885 | NA | NA | SCARB1 |
| Central carbon metabolism in cancer | 1 | 209 | 0.478354184468 | 0.676823488909 | Cluster-11054.51840 | K05093 | NA | NA | FGFR2 |
| Leukocyte transendothelial migration | 2 | 521 | 0.481202069421 | 0.676823488909 | Cluster-11054.44184 Cluster-11054.55722 | K05719 K05719 | NA | NA | ITGB1 ITGB1 |
| Insulin secretion | 1 | 212 | 0.483188300575 | 0.676823488909 | Cluster-11054.44073 | K04962 | NA | NA | RYR2 |
| Ras signaling pathway | 2 | 525 | 0.485182090737 | 0.676823488909 | Cluster-11054.48563 Cluster-11054.51840 | K17633 K05093 | NA | NA | RASAL2 FGFR2 |
| Circadian entrainment | 1 | 215 | 0.487977815094 | 0.676823488909 | Cluster-11054.44073 | K04962 | NA | NA | RYR2 |
| Hepatitis C | 1 | 217 | 0.491146248589 | 0.676823488909 | Cluster-11054.31994 | K13885 | NA | NA | SCARB1 |
| Serotonergic synapse | 1 | 221 | 0.497424674423 | 0.677216725419 | Cluster-11054.40552 | K00461 | NA | NA | ALOX5 |
| ErbB signaling pathway | 1 | 228 | 0.508226900707 | 0.683686187856 | Cluster-11054.23418 | K07365 | NA | NA | NCK |
| Apoptosis - fly | 1 | 244 | 0.532057533149 | 0.707323544069 | Cluster-11054.54952 | K10798 | NA | NA | PARP |
| Antigen processing and presentation | 1 | 261 | 0.556118866551 | 0.717281880841 | Cluster-11054.50465 | K03283 | NA | NA | HSPA1\_8 |
| Signaling pathways regulating pluripotency of stem cells | 1 | 264 | 0.560235259417 | 0.717281880841 | Cluster-11054.51840 | K05093 | NA | NA | FGFR2 |
| T cell receptor signaling pathway | 1 | 267 | 0.564313645696 | 0.717281880841 | Cluster-11054.23418 | K07365 | NA | NA | NCK |
| Lysine degradation | 1 | 269 | 0.567011627772 | 0.717281880841 | Cluster-11054.22506 | K00471 | NA | NA | E1.14.11.1 |
| Platelet activation | 2 | 617 | 0.571286453767 | 0.717281880841 | Cluster-11054.44184 Cluster-11054.55722 | K05719 K05719 | NA | NA | ITGB1 ITGB1 |
| MAPK signaling pathway | 2 | 635 | 0.586870421948 | 0.728751183299 | Cluster-11054.51840 Cluster-11054.50465 | K05093 K03283 | NA | NA | FGFR2 HSPA1\_8 |
| Pathways in cancer | 3 | 1007 | 0.605317153224 | 0.737927222981 | Cluster-11054.44184 Cluster-11054.55722 Cluster-11054.51840 | K05719 K05719 K05093 | NA | NA | ITGB1 ITGB1 FGFR2 |
| Tight junction | 3 | 1010 | 0.607320634843 | 0.737927222981 | Cluster-11054.46692 Cluster-11054.54104 Cluster-11054.48110 | K03097 K03097 K03097 | NA | NA | CSNK2A CSNK2A CSNK2A |
| Protein processing in endoplasmic reticulum | 2 | 723 | 0.657004896767 | 0.787811218898 | Cluster-11054.50465 Cluster-11054.48334 | K03283 K14012 | NA | NA | HSPA1\_8 SHP1, UBX1, NSFL1C |
| mRNA surveillance pathway | 1 | 349 | 0.66231916633 | 0.787811218898 | Cluster-11054.34236 | K14416 | NA | NA | HBS1 |
| Pyrimidine metabolism | 1 | 391 | 0.703670646776 | 0.81873644846 | Cluster-11054.40917 | K00962 | NA | NA | pnp, PNPT1 |
| Focal adhesion | 2 | 794 | 0.706414441536 | 0.81873644846 | Cluster-11054.44184 Cluster-11054.55722 | K05719 K05719 | NA | NA | ITGB1 ITGB1 |
| Sphingolipid signaling pathway | 1 | 398 | 0.710054619019 | 0.81873644846 | Cluster-11054.66341 | K12348 | NA | NA | ASAH1 |
| Pancreatic secretion | 1 | 416 | 0.725848765229 | 0.828494045161 | Cluster-11054.44073 | K04962 | NA | NA | RYR2 |
| Proteoglycans in cancer | 2 | 854 | 0.743432200374 | 0.837689862957 | Cluster-11054.44184 Cluster-11054.55722 | K05719 K05719 | NA | NA | ITGB1 ITGB1 |
| cAMP signaling pathway | 1 | 444 | 0.748731647421 | 0.837689862957 | Cluster-11054.44073 | K04962 | NA | NA | RYR2 |
| Oxidative phosphorylation | 1 | 455 | 0.757191880043 | 0.838849827891 | Cluster-11054.37506 | K00237 | NA | NA | SDHD, SDH4 |
| Endocytosis | 2 | 911 | 0.774840240269 | 0.850067448062 | Cluster-11054.51840 Cluster-11054.50465 | K05093 K03283 | NA | NA | FGFR2 HSPA1\_8 |
| Parkinson's disease | 1 | 514 | 0.797957449712 | 0.867011459783 | Cluster-11054.37506 | K00237 | NA | NA | SDHD, SDH4 |
| Alzheimer's disease | 1 | 600 | 0.845482340795 | 0.897690205316 | Cluster-11054.37506 | K00237 | NA | NA | SDHD, SDH4 |
| Viral carcinogenesis | 1 | 626 | 0.857524354675 | 0.897690205316 | Cluster-11054.24956 | K16174 | NA | NA | MRPS18B, MRPS18-2 |
| Lysosome | 1 | 639 | 0.86319022025 | 0.897690205316 | Cluster-11054.66341 | K12348 | NA | NA | ASAH1 |
| Oxytocin signaling pathway | 1 | 640 | 0.863616648184 | 0.897690205316 | Cluster-11054.44073 | K04962 | NA | NA | RYR2 |
| Huntington's disease | 1 | 659 | 0.871471954546 | 0.897690205316 | Cluster-11054.37506 | K00237 | NA | NA | SDHD, SDH4 |
| Cardiac muscle contraction | 1 | 665 | 0.873857721989 | 0.897690205316 | Cluster-11054.44073 | K04962 | NA | NA | RYR2 |
| Influenza A | 1 | 724 | 0.895092005117 | 0.904725161637 | Cluster-11054.50465 | K03283 | NA | NA | HSPA1\_8 |
| Apoptosis | 1 | 729 | 0.896718744277 | 0.904725161637 | Cluster-11054.54952 | K10798 | NA | NA | PARP |
| Adrenergic signaling in cardiomyocytes | 1 | 832 | 0.925165267615 | 0.925165267615 | Cluster-11054.44073 | K04962 | NA | NA | RYR2 |
