# Supplementary material for: Screening of the candidate genes related to low-temperature tolerance of Fenneropenaeus chinensis based on high-throughput transcriptome sequencing
Source: PLoS One. 2019 Apr 8;14(4):e0211182. doi: 10.1371/journal.pone.0211182 (PMC6453463; doi:10.1371/journal.pone.0211182)
Supplement: S1 File — (ZIP) [file pone.0211182.s001.zip › Fc-low-tem-SNP-GO/DEG_KEGGenrichment/LvsN_up.DEG_enriched_KEGG_pathway_scatterplot.pdf]

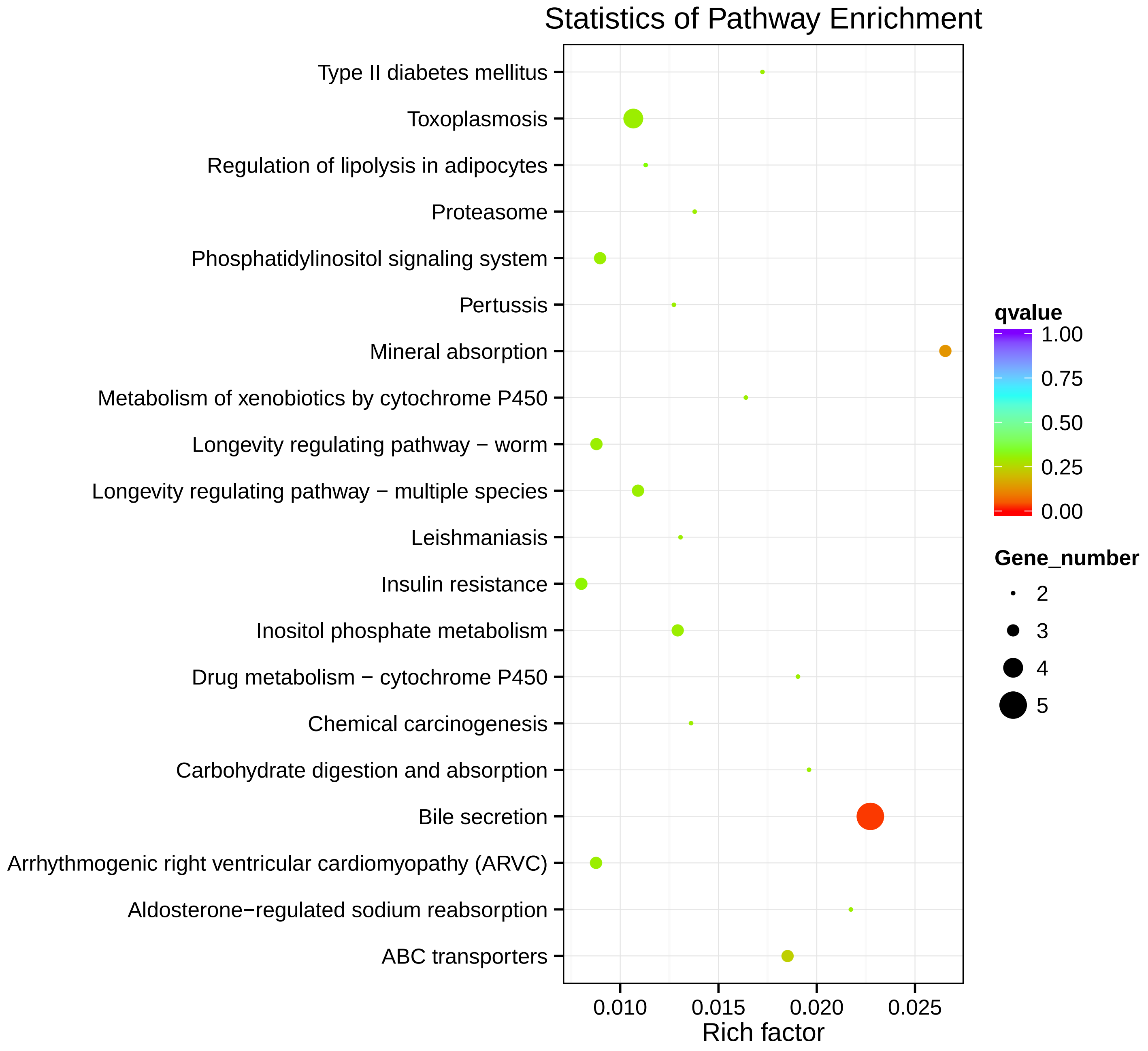

Statistics of Pathway Enrichment
Type II diabetes mellitus
Toxoplasmosis
Regulation of lipolysis in adipocytes
Proteasome
Phosphatidylinositol signaling system
Pertussis
Mineral absorption
Metabolism of xenobiotics by cytochrome P450
Longevity regulating pathway − worm
Longevity regulating pathway − multiple species
Leishmaniasis
Insulin resistance
Inositol phosphate metabolism
Drug metabolism − cytochrome P450
Chemical carcinogenesis
Carbohydrate digestion and absorption
Bile secretion
Arrhythmogenic right ventricular cardiomyopathy (ARVC)
Aldosterone−regulated sodium reabsorption
ABC transporters
0.010
0.015
0.020
0.025
Rich factor
qvalue
1.00
0.75
0.50
0.25
0.00
Gene_number
2
3
4
5
