# Supplementary material for: Screening of the candidate genes related to low-temperature tolerance of Fenneropenaeus chinensis based on high-throughput transcriptome sequencing
Source: PLoS One. 2019 Apr 8;14(4):e0211182. doi: 10.1371/journal.pone.0211182 (PMC6453463; doi:10.1371/journal.pone.0211182)
Supplement: S1 File — (ZIP) [file pone.0211182.s001.zip › Fc-low-tem-SNP-GO/DEG_KEGGenrichment/LvsN_up_kegg_web/LvsN_up.DEG_enriched_KEGG_pathway_API.html]

Pathway Enrichment


  

# The most enriched pathway terms

Statistic method: hypergeometric test

FDR correction method: Benjamini and Hochberg

| Term | Sample number | Background number | P-value | Corrected P-value | UniGenes | KO | Entrez ID | Ensembl ID | Gene name |
| --- | --- | --- | --- | --- | --- | --- | --- | --- | --- |
| Bile secretion | 5 | 220 | 0.000223155832861 | 0.0214229599546 | Cluster-11054.55919 Cluster-11054.55918 Cluster-11054.54514 Cluster-11054.54276 Cluster-11054.54510 | K05666 K05667 K14158 K05667 K14158 | NA | NA | ABCC2 ABCC3 SLC5A1, SGLT1 ABCC3 SLC5A1, SGLT1 |
| Mineral absorption | 3 | 113 | 0.00280362922221 | 0.134574202666 | Cluster-11054.54514 Cluster-11054.47412 Cluster-11054.54510 | K14158 K07213 K14158 | NA | NA | SLC5A1, SGLT1 ATOX1, ATX1, copZ, golB SLC5A1, SGLT1 |
| ABC transporters | 3 | 162 | 0.00746714592329 | 0.238948669545 | Cluster-11054.55919 Cluster-11054.55918 Cluster-11054.54276 | K05666 K05667 K05667 | NA | NA | ABCC2 ABCC3 ABCC3 |
| Toxoplasmosis | 4 | 375 | 0.0134290695107 | 0.299062826799 | Cluster-11054.44184 Cluster-11054.55722 Cluster-11054.40552 Cluster-11054.50465 | K05719 K05719 K00461 K03283 | NA | NA | ITGB1 ITGB1 ALOX5 HSPA1\_8 |
| Inositol phosphate metabolism | 3 | 232 | 0.0192204071119 | 0.299062826799 | Cluster-11054.40113 Cluster-11054.66262 Cluster-11054.40114 | K00911 K01099 K00911 | NA | NA | ITPK E3.1.3.36 ITPK |
| Aldosterone-regulated sodium reabsorption | 2 | 92 | 0.0215693489446 | 0.299062826799 | Cluster-11054.44711 Cluster-11054.40313 | K16172 K16172 | NA | NA | IRS1 IRS1 |
| Carbohydrate digestion and absorption | 2 | 102 | 0.0260234600784 | 0.299062826799 | Cluster-11054.54514 Cluster-11054.54510 | K14158 K14158 | NA | NA | SLC5A1, SGLT1 SLC5A1, SGLT1 |
| Drug metabolism - cytochrome P450 | 2 | 105 | 0.0274262979931 | 0.299062826799 | Cluster-11054.39485 Cluster-11054.39487 | K00799 K00799 | NA | NA | GST, gst GST, gst |
| Longevity regulating pathway - multiple species | 3 | 275 | 0.029615424052 | 0.299062826799 | Cluster-11054.44711 Cluster-11054.40313 Cluster-11054.50465 | K16172 K16172 K03283 | NA | NA | IRS1 IRS1 HSPA1\_8 |
| Type II diabetes mellitus | 2 | 116 | 0.0328216420256 | 0.299062826799 | Cluster-11054.44711 Cluster-11054.40313 | K16172 K16172 | NA | NA | IRS1 IRS1 |
| Metabolism of xenobiotics by cytochrome P450 | 2 | 122 | 0.0359250864308 | 0.299062826799 | Cluster-11054.39485 Cluster-11054.39487 | K00799 K00799 | NA | NA | GST, gst GST, gst |
| Phosphatidylinositol signaling system | 3 | 334 | 0.0477990782426 | 0.299062826799 | Cluster-11054.40113 Cluster-11054.66262 Cluster-11054.40114 | K00911 K01099 K00911 | NA | NA | ITPK E3.1.3.36 ITPK |
| Proteasome | 2 | 145 | 0.0487944799633 | 0.299062826799 | Cluster-11054.44996 Cluster-11054.50114 | K03036 K03029 | NA | NA | PSMD11, RPN6 PSMD4, RPN10 |
| Chemical carcinogenesis | 2 | 147 | 0.0499821917657 | 0.299062826799 | Cluster-11054.39485 Cluster-11054.39487 | K00799 K00799 | NA | NA | GST, gst GST, gst |
| Longevity regulating pathway - worm | 3 | 341 | 0.0502485932408 | 0.299062826799 | Cluster-11054.54952 Cluster-11054.44711 Cluster-11054.40313 | K10798 K16172 K16172 | NA | NA | PARP IRS1 IRS1 |
| Arrhythmogenic right ventricular cardiomyopathy (ARVC) | 3 | 342 | 0.050603446269 | 0.299062826799 | Cluster-11054.44184 Cluster-11054.55722 Cluster-11054.44073 | K05719 K05719 K04962 | NA | NA | ITGB1 ITGB1 RYR2 |
| Leishmaniasis | 2 | 153 | 0.0536072356218 | 0.299062826799 | Cluster-11054.44184 Cluster-11054.55722 | K05719 K05719 | NA | NA | ITGB1 ITGB1 |
| Pertussis | 2 | 157 | 0.0560742800247 | 0.299062826799 | Cluster-11054.44184 Cluster-11054.55722 | K05719 K05719 | NA | NA | ITGB1 ITGB1 |
| Insulin resistance | 3 | 374 | 0.0625953576523 | 0.316271280769 | Cluster-11054.52075 Cluster-11054.44711 Cluster-11054.40313 | K15719 K16172 K16172 | NA | NA | NCOAT, MGEA5 IRS1 IRS1 |
| Regulation of lipolysis in adipocytes | 2 | 177 | 0.0689775418403 | 0.331092200833 | Cluster-11054.44711 Cluster-11054.40313 | K16172 K16172 | NA | NA | IRS1 IRS1 |
| Epstein-Barr virus infection | 4 | 668 | 0.0789106731827 | 0.345718049378 | Cluster-11054.54104 Cluster-11054.44996 Cluster-11054.50114 Cluster-11054.50465 | K03097 K03036 K03029 K03283 | NA | NA | CSNK2A PSMD11, RPN6 PSMD4, RPN10 HSPA1\_8 |
| Cell adhesion molecules (CAMs) | 2 | 192 | 0.0792270529825 | 0.345718049378 | Cluster-11054.44184 Cluster-11054.55722 | K05719 K05719 | NA | NA | ITGB1 ITGB1 |
| Glutathione metabolism | 2 | 199 | 0.0841630872242 | 0.349394016273 | Cluster-11054.39485 Cluster-11054.39487 | K00799 K00799 | NA | NA | GST, gst GST, gst |
| Calcium signaling pathway | 3 | 439 | 0.0905345667233 | 0.349394016273 | Cluster-11054.40113 Cluster-11054.44073 Cluster-11054.40114 | K00911 K04962 K00911 | NA | NA | ITPK RYR2 ITPK |
| PI3K-Akt signaling pathway | 4 | 721 | 0.0974018436205 | 0.349394016273 | Cluster-11054.44184 Cluster-11054.55722 Cluster-11054.44711 Cluster-11054.40313 | K05719 K05719 K16172 K16172 | NA | NA | ITGB1 ITGB1 IRS1 IRS1 |
| ECM-receptor interaction | 2 | 220 | 0.0995004041747 | 0.349394016273 | Cluster-11054.44184 Cluster-11054.55722 | K05719 K05719 | NA | NA | ITGB1 ITGB1 |
| Legionellosis | 2 | 222 | 0.100999645951 | 0.349394016273 | Cluster-11054.34236 Cluster-11054.50465 | K14416 K03283 | NA | NA | HBS1 HSPA1\_8 |
| mTOR signaling pathway | 2 | 224 | 0.102505190155 | 0.349394016273 | Cluster-11054.44711 Cluster-11054.40313 | K16172 K16172 | NA | NA | IRS1 IRS1 |
| Small cell lung cancer | 2 | 235 | 0.110894189423 | 0.349394016273 | Cluster-11054.44184 Cluster-11054.55722 | K05719 K05719 | NA | NA | ITGB1 ITGB1 |
| Adipocytokine signaling pathway | 2 | 240 | 0.114765342718 | 0.349394016273 | Cluster-11054.44711 Cluster-11054.40313 | K16172 K16172 | NA | NA | IRS1 IRS1 |
| Glycosphingolipid biosynthesis - globo series | 1 | 51 | 0.117226511198 | 0.349394016273 | Cluster-11054.57109 | K00718 | NA | NA | FUT1\_2 |
| Selenocompound metabolism | 1 | 52 | 0.119341474241 | 0.349394016273 | Cluster-11054.54624 | K00548 | NA | NA | metH, MTR |
| Non-alcoholic fatty liver disease (NAFLD) | 3 | 499 | 0.120104193094 | 0.349394016273 | Cluster-11054.37506 Cluster-11054.44711 Cluster-11054.40313 | K00237 K16172 K16172 | NA | NA | SDHD, SDH4 IRS1 IRS1 |
| RNA degradation | 2 | 262 | 0.132187725702 | 0.373235931393 | Cluster-11054.37559 Cluster-11054.40917 | K12604 K00962 | NA | NA | CNOT1, NOT1 pnp, PNPT1 |
| Hypertrophic cardiomyopathy (HCM) | 3 | 544 | 0.144302431887 | 0.391811130201 | Cluster-11054.44184 Cluster-11054.55722 Cluster-11054.44073 | K05719 K05719 K04962 | NA | NA | ITGB1 ITGB1 RYR2 |
| Glycosphingolipid biosynthesis - lacto and neolacto series | 1 | 66 | 0.148427339574 | 0.391811130201 | Cluster-11054.57109 | K00718 | NA | NA | FUT1\_2 |
| Dilated cardiomyopathy (DCM) | 3 | 556 | 0.151010539765 | 0.391811130201 | Cluster-11054.44184 Cluster-11054.55722 Cluster-11054.44073 | K05719 K05719 K04962 | NA | NA | ITGB1 ITGB1 RYR2 |
| Measles | 2 | 305 | 0.167743723658 | 0.423773617662 | Cluster-11054.54104 Cluster-11054.50465 | K03097 K03283 | NA | NA | CSNK2A HSPA1\_8 |
| Longevity regulating pathway | 2 | 319 | 0.179652084352 | 0.433200908596 | Cluster-11054.44711 Cluster-11054.40313 | K16172 K16172 | NA | NA | IRS1 IRS1 |
| One carbon pool by folate | 1 | 82 | 0.180500378582 | 0.433200908596 | Cluster-11054.54624 | K00548 | NA | NA | metH, MTR |
| Glycosylphosphatidylinositol(GPI)-anchor biosynthesis | 1 | 91 | 0.198010626723 | 0.463634638181 | Cluster-11054.36172 | K05290 | NA | NA | PIGK |
| FoxO signaling pathway | 2 | 352 | 0.208175713129 | 0.475830201438 | Cluster-11054.44711 Cluster-11054.40313 | K16172 K16172 | NA | NA | IRS1 IRS1 |
| Neurotrophin signaling pathway | 2 | 358 | 0.213414986514 | 0.476461365239 | Cluster-11054.44711 Cluster-11054.40313 | K16172 K16172 | NA | NA | IRS1 IRS1 |
| Axon guidance | 2 | 378 | 0.230962362986 | 0.501914152126 | Cluster-11054.44184 Cluster-11054.55722 | K05719 K05719 | NA | NA | ITGB1 ITGB1 |
| Spliceosome | 3 | 696 | 0.235272258809 | 0.501914152126 | Cluster-11054.59082 Cluster-11054.50465 Cluster-11054.18182 | K12896 K03283 K12850 | NA | NA | SFRS7 HSPA1\_8 PRPF38B |
| AMPK signaling pathway | 2 | 402 | 0.252133128441 | 0.526190876746 | Cluster-11054.44711 Cluster-11054.40313 | K16172 K16172 | NA | NA | IRS1 IRS1 |
| Shigellosis | 2 | 425 | 0.272469040072 | 0.549862136533 | Cluster-11054.44184 Cluster-11054.55722 | K05719 K05719 | NA | NA | ITGB1 ITGB1 |
| Citrate cycle (TCA cycle) | 1 | 133 | 0.274931068267 | 0.549862136533 | Cluster-11054.37506 | K00237 | NA | NA | SDHD, SDH4 |
| Ovarian steroidogenesis | 1 | 140 | 0.287017905179 | 0.56232079382 | Cluster-11054.40552 | K00461 | NA | NA | ALOX5 |
| Insulin signaling pathway | 2 | 460 | 0.303362141164 | 0.565675054382 | Cluster-11054.44711 Cluster-11054.40313 | K16172 K16172 | NA | NA | IRS1 IRS1 |
| Arachidonic acid metabolism | 1 | 153 | 0.308935840515 | 0.565675054382 | Cluster-11054.40552 | K00461 | NA | NA | ALOX5 |
| Base excision repair | 1 | 156 | 0.31389791849 | 0.565675054382 | Cluster-11054.54952 | K10798 | NA | NA | PARP |
| NF-kappa B signaling pathway | 1 | 160 | 0.32045895687 | 0.565675054382 | Cluster-11054.54104 | K03097 | NA | NA | CSNK2A |
| cGMP-PKG signaling pathway | 2 | 487 | 0.327044169502 | 0.565675054382 | Cluster-11054.44711 Cluster-11054.40313 | K16172 K16172 | NA | NA | IRS1 IRS1 |
| Bacterial invasion of epithelial cells | 2 | 496 | 0.334893916491 | 0.565675054382 | Cluster-11054.44184 Cluster-11054.55722 | K05719 K05719 | NA | NA | ITGB1 ITGB1 |
| Herpes simplex infection | 2 | 501 | 0.339243597445 | 0.565675054382 | Cluster-11054.59082 Cluster-11054.54104 | K12896 K03097 | NA | NA | SFRS7 CSNK2A |
| Pathogenic Escherichia coli infection | 2 | 506 | 0.343584676525 | 0.565675054382 | Cluster-11054.44184 Cluster-11054.55722 | K05719 K05719 | NA | NA | ITGB1 ITGB1 |
| MicroRNAs in cancer | 2 | 508 | 0.345318607139 | 0.565675054382 | Cluster-11054.44711 Cluster-11054.40313 | K16172 K16172 | NA | NA | IRS1 IRS1 |
| Sphingolipid metabolism | 1 | 177 | 0.347654460506 | 0.565675054382 | Cluster-11054.66341 | K12348 | NA | NA | ASAH1 |
| Leukocyte transendothelial migration | 2 | 521 | 0.356552244106 | 0.57048359057 | Cluster-11054.44184 Cluster-11054.55722 | K05719 K05719 | NA | NA | ITGB1 ITGB1 |
| Cysteine and methionine metabolism | 1 | 187 | 0.363143408557 | 0.57150438068 | Cluster-11054.54624 | K00548 | NA | NA | metH, MTR |
| Insulin secretion | 1 | 212 | 0.400284526691 | 0.616524445426 | Cluster-11054.44073 | K04962 | NA | NA | RYR2 |
| Circadian entrainment | 1 | 215 | 0.404594167311 | 0.616524445426 | Cluster-11054.44073 | K04962 | NA | NA | RYR2 |
| Serotonergic synapse | 1 | 221 | 0.413121285374 | 0.619681928061 | Cluster-11054.40552 | K00461 | NA | NA | ALOX5 |
| Platelet activation | 2 | 617 | 0.436998751932 | 0.645413541315 | Cluster-11054.44184 Cluster-11054.55722 | K05719 K05719 | NA | NA | ITGB1 ITGB1 |
| Apoptosis - fly | 1 | 244 | 0.444698573105 | 0.646834288153 | Cluster-11054.54952 | K10798 | NA | NA | PARP |
| Antigen processing and presentation | 1 | 261 | 0.466947094806 | 0.666426184952 | Cluster-11054.50465 | K03283 | NA | NA | HSPA1\_8 |
| Ribosome biogenesis in eukaryotes | 1 | 265 | 0.472051881007 | 0.666426184952 | Cluster-11054.54104 | K03097 | NA | NA | CSNK2A |
| Protein processing in endoplasmic reticulum | 2 | 723 | 0.519158790806 | 0.712999673472 | Cluster-11054.50465 Cluster-11054.48334 | K03283 K14012 | NA | NA | HSPA1\_8 SHP1, UBX1, NSFL1C |
| Rap1 signaling pathway | 2 | 724 | 0.51989559524 | 0.712999673472 | Cluster-11054.44184 Cluster-11054.55722 | K05719 K05719 | NA | NA | ITGB1 ITGB1 |
| Phagosome | 2 | 744 | 0.534473878792 | 0.721247049096 | Cluster-11054.44184 Cluster-11054.55722 | K05719 K05719 | NA | NA | ITGB1 ITGB1 |
| Regulation of actin cytoskeleton | 2 | 753 | 0.540935286822 | 0.721247049096 | Cluster-11054.44184 Cluster-11054.55722 | K05719 K05719 | NA | NA | ITGB1 ITGB1 |
| mRNA surveillance pathway | 1 | 349 | 0.568701746082 | 0.729266291633 | Cluster-11054.34236 | K14416 | NA | NA | HBS1 |
| Focal adhesion | 2 | 794 | 0.569583435729 | 0.729266291633 | Cluster-11054.44184 Cluster-11054.55722 | K05719 K05719 | NA | NA | ITGB1 ITGB1 |
| Estrogen signaling pathway | 1 | 350 | 0.569739290338 | 0.729266291633 | Cluster-11054.50465 | K03283 | NA | NA | HSPA1\_8 |
| Proteoglycans in cancer | 2 | 854 | 0.609151285671 | 0.759050776623 | Cluster-11054.44184 Cluster-11054.55722 | K05719 K05719 | NA | NA | ITGB1 ITGB1 |
| Pyrimidine metabolism | 1 | 391 | 0.610208064991 | 0.759050776623 | Cluster-11054.40917 | K00962 | NA | NA | pnp, PNPT1 |
| Sphingolipid signaling pathway | 1 | 398 | 0.616728756006 | 0.759050776623 | Cluster-11054.66341 | K12348 | NA | NA | ASAH1 |
| Pancreatic secretion | 1 | 416 | 0.63300247872 | 0.769218201989 | Cluster-11054.44073 | K04962 | NA | NA | RYR2 |
| cAMP signaling pathway | 1 | 444 | 0.656961910579 | 0.788354292695 | Cluster-11054.44073 | K04962 | NA | NA | RYR2 |
| Oxidative phosphorylation | 1 | 455 | 0.665943159696 | 0.789265967047 | Cluster-11054.37506 | K00237 | NA | NA | SDHD, SDH4 |
| Adherens junction | 1 | 467 | 0.675474409881 | 0.790799309129 | Cluster-11054.54104 | K03097 | NA | NA | CSNK2A |
| Pathways in cancer | 2 | 1007 | 0.697438001428 | 0.80667527876 | Cluster-11054.44184 Cluster-11054.55722 | K05719 K05719 | NA | NA | ITGB1 ITGB1 |
| Parkinson's disease | 1 | 514 | 0.710271743893 | 0.811739135878 | Cluster-11054.37506 | K00237 | NA | NA | SDHD, SDH4 |
| Alzheimer's disease | 1 | 600 | 0.764616356405 | 0.842743010144 | Cluster-11054.37506 | K00237 | NA | NA | SDHD, SDH4 |
| Purine metabolism | 1 | 609 | 0.76968139113 | 0.842743010144 | Cluster-11054.40917 | K00962 | NA | NA | pnp, PNPT1 |
| MAPK signaling pathway | 1 | 635 | 0.783713024454 | 0.842743010144 | Cluster-11054.50465 | K03283 | NA | NA | HSPA1\_8 |
| Lysosome | 1 | 639 | 0.785794951018 | 0.842743010144 | Cluster-11054.66341 | K12348 | NA | NA | ASAH1 |
| Oxytocin signaling pathway | 1 | 640 | 0.786312311672 | 0.842743010144 | Cluster-11054.44073 | K04962 | NA | NA | RYR2 |
| Huntington's disease | 1 | 659 | 0.795909449176 | 0.842743010144 | Cluster-11054.37506 | K00237 | NA | NA | SDHD, SDH4 |
| Cardiac muscle contraction | 1 | 665 | 0.798850145032 | 0.842743010144 | Cluster-11054.44073 | K04962 | NA | NA | RYR2 |
| Influenza A | 1 | 724 | 0.825614386854 | 0.854413086963 | Cluster-11054.50465 | K03283 | NA | NA | HSPA1\_8 |
| Apoptosis | 1 | 729 | 0.827712677995 | 0.854413086963 | Cluster-11054.54952 | K10798 | NA | NA | PARP |
| Adrenergic signaling in cardiomyocytes | 1 | 832 | 0.86576329331 | 0.884183788912 | Cluster-11054.44073 | K04962 | NA | NA | RYR2 |
| Endocytosis | 1 | 911 | 0.889174815558 | 0.898534550459 | Cluster-11054.50465 | K03283 | NA | NA | HSPA1\_8 |
| Tight junction | 1 | 1010 | 0.912862482658 | 0.912862482658 | Cluster-11054.54104 | K03097 | NA | NA | CSNK2A |
