# Supplementary material for: Screening of the candidate genes related to low-temperature tolerance of Fenneropenaeus chinensis based on high-throughput transcriptome sequencing
Source: PLoS One. 2019 Apr 8;14(4):e0211182. doi: 10.1371/journal.pone.0211182 (PMC6453463; doi:10.1371/journal.pone.0211182)
Supplement: S1 File — (ZIP) [file pone.0211182.s001.zip › Fc-low-tem-SNP-GO/DEG_KEGGenrichment/LvsN_up_kegg_web/src/ko04976.html]

ko04976


- K05666

- Up regulated genes

Cluster-11054.55919(Inf)

- K05667

- Up regulated genes

Cluster-11054.55918(Inf) Cluster-11054.54276(Inf)

- K05667

- Up regulated genes

Cluster-11054.55918(Inf) Cluster-11054.54276(Inf)

- K14158

- Up regulated genes

Cluster-11054.54514(Inf) Cluster-11054.54510(Inf)

Close
