# Supplementary material for: Screening of the candidate genes related to low-temperature tolerance of Fenneropenaeus chinensis based on high-throughput transcriptome sequencing
Source: PLoS One. 2019 Apr 8;14(4):e0211182. doi: 10.1371/journal.pone.0211182 (PMC6453463; doi:10.1371/journal.pone.0211182)
Supplement: S1 File — (ZIP) [file pone.0211182.s001.zip › Fc-low-tem-SNP-GO/DEG_PPI/CytoscapeQuickStart.pdf]

Novogene 诺禾致源

Cytoscape Quick Start

Cytoscape

Network Data Integration, Analysis, and Visualization in a Box

Cytoscape Quick Start

一、 软件安装

1. 安装 Java Runtime Environment (JRE)

需要 Java 8 或更高的版本

推荐使用 64 bit Java Runtime Environment (JRE)

下载地址：

http://www.oracle.com/technetwork/java/javase/downloads/jdk8-downloads-2133151.html

下载方法：点击选择 Acecept License Agreement 下载 64 版本 java

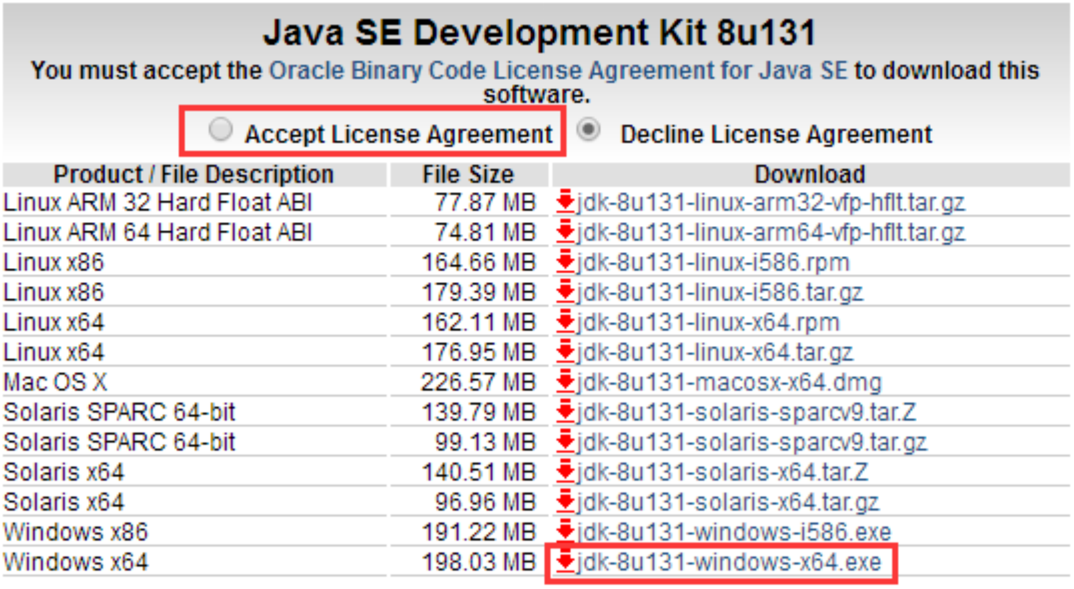

Java SE Development Kit 8u131

You must accept the Oracle Binary Code License Agreement for Java SE to download this software.

Accept License Agreement   Decline License Agreement

Product / File Description	File Size	Download
Linux ARM 32 Hard Float ABI	77.87 MB	jdk-8u131-linux-arm32-vfp-hflt.tar.gz
Linux ARM 64 Hard Float ABI	74.81 MB	jdk-8u131-linux-arm64-vfp-hflt.tar.gz
Linux x86	164.66 MB	jdk-8u131-linux-i586.rpm
Linux x86	179.39 MB	jdk-8u131-linux-i586.tar.gz
Linux x64	162.11 MB	jdk-8u131-linux-x64.rpm
Linux x64	176.95 MB	jdk-8u131-linux-x64.tar.gz
Mac OS X	226.57 MB	jdk-8u131-macosx-x64.dmg
Solaris SPARC 64-bit	139.79 MB	jdk-8u131-solaris-sparcv9.tar.Z
Solaris SPARC 64-bit	99.13 MB	jdk-8u131-solaris-sparcv9.tar.gz
Solaris x64	140.51 MB	jdk-8u131-solaris-x64.tar.Z
Solaris x64	96.96 MB	jdk-8u131-solaris-x64.tar.gz
Windows x86	191.22 MB	jdk-8u131-windows-i586.exe
Windows x64	198.03 MB	jdk-8u131-windows-x64.exe

2. 安装 Cytoscape 3.5.1

下载地址：

http://www.cytoscape.org/download.html

二、 基本概念

网络（network）：整个网络图，我们的数据形成的网络图。

节点(node)：即相互作用的分子，可以是蛋白、基因等。

边（edge）：连接两个节点的线，代表了节点与节点的相互作用。

度（degree）：表示与此节点相互作用的节点的个数，度的大小与此节点的核心性（BetweennessCentrality）成正比，即依赖此节点的通路越多，它的核心性越大，度也就越大。

三、 输入文件格式

Cytoscape 软件导入文件格式为 txt 格式（Tab 为默认分隔符）。首先导入 network 数据，至少包含 2 列信息，即网络中节点与节点的对应关系：相互作用的一对基因 ID 或基因 name。同时，节点间的相互作用关系（例如相关性系数、互作得分值等）可以作为 interaction 数据一同导入进来。如果需要展示更为丰富的内容，则需要通过导入 Table 对 Network 中的 Nodes 和 Eges 进行注释，从而达到我们想要的结果。通过 Table 导入的属性文件格式为：第 1 列为 key(network 文件中的基因 ID 或基因 name)，其他列为对基因的描述信息（例如 gene type，上下调信息，差异显著性等）。

四、 操作步骤演示

1. 打开 cytoscape 软件

界面如下图，导入 network 文件（以本公司提供的 PPI 分析结果文件为例）：

File —> Import —> Network —> File 会弹出导入文件的对话框，在对话框中做设置如下：

（1）Source Node: column 1 选择互作初始节点列（原节点）。

（2）Target Node: column 2 选择互作目标节点列（靶向的节点）。

（3）Data Type: 选择 ab（蛋白蛋白相互作用）。

（4）单击其他要输入的列，可以选择添加边、原节点和目标节点的属性。

（5）选择 Advanced，根据实际情况勾选 Coumn Names，选中该选项则默认输入文件

的第 1 行为表头信息。

（6）单击 OK。

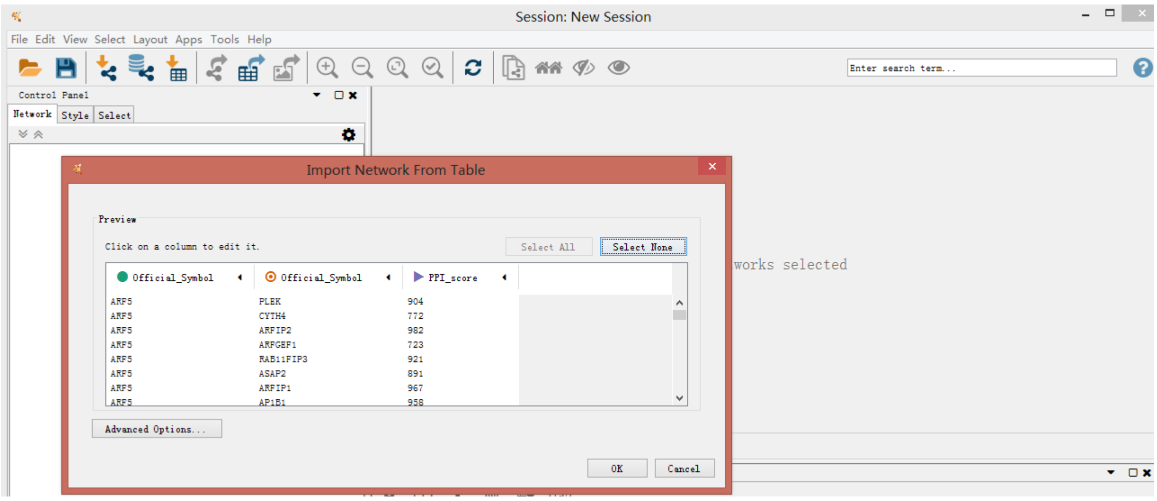

2. 数据导入完成

导入数据后，会出现如下图的界面：包括菜单栏和快捷菜单栏、控制面板（Control Panel，可对网络进行个性化操作），网络窗口（一个框代表一个节点，边用于连接相互作用的节点），表格面板（Table Panel，显示节点和边的信息）。

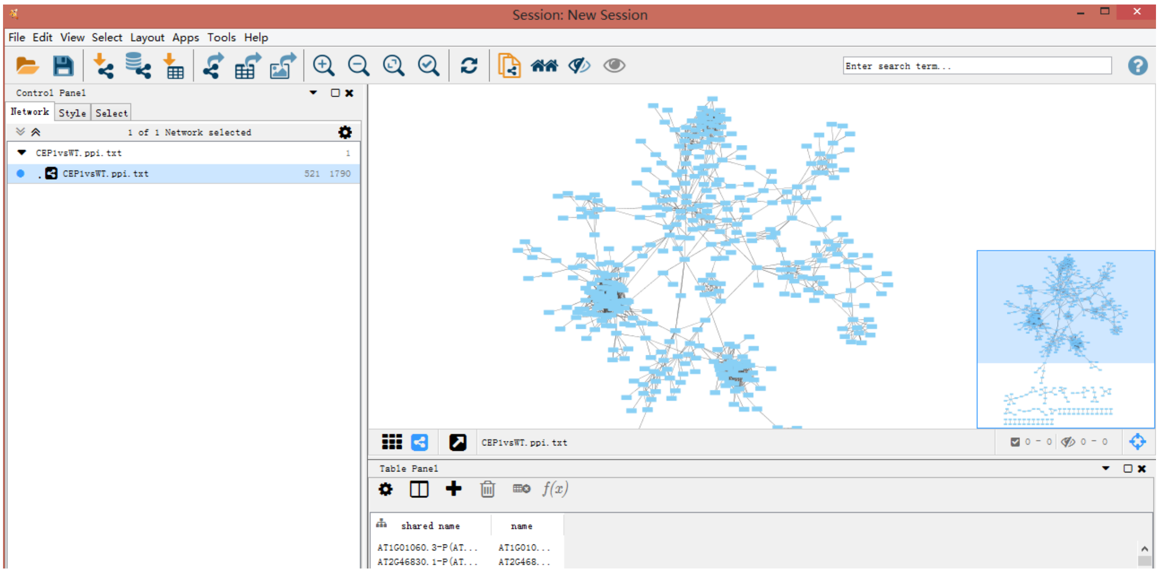

3. 对网络的个性化操作

1）节点的选择

方法 1：按住 shift 键，点击鼠标左键，即可手动将需要展示的节点选中。

方法 2：选中目标节点，然后选择 select—>Nodes—>fisrt Neighbors of Selected Nodes —>Undircted(实际情况如果是有方向性的，需要选择 Incoming/Outgoing)，与选中节点第一相邻的节点即被选择出来了。

节点选择后，点击 file—>New—>Network—>from selected notes, all edges 选择出来的节点即被保存为新的 network。

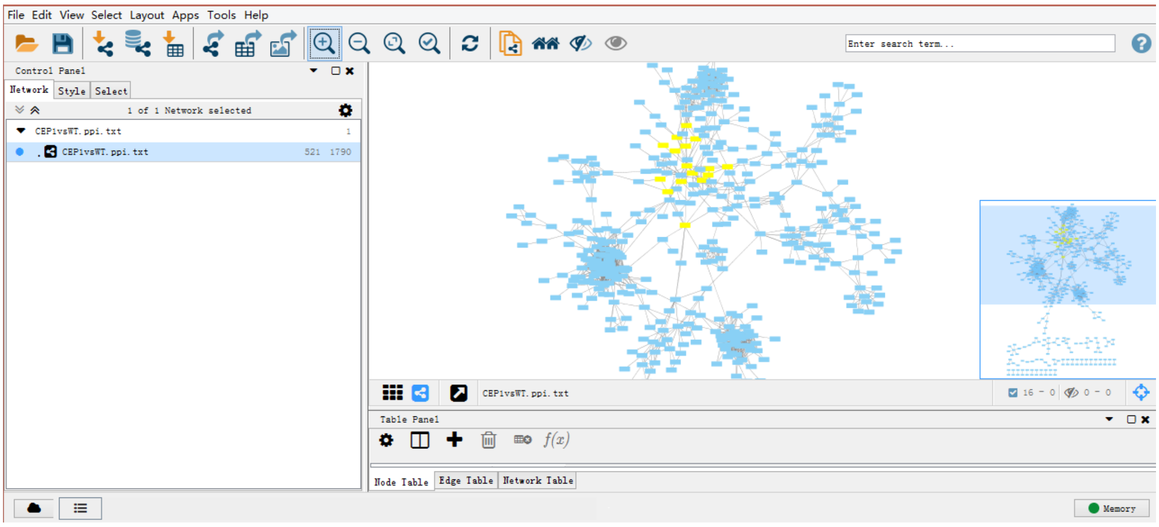

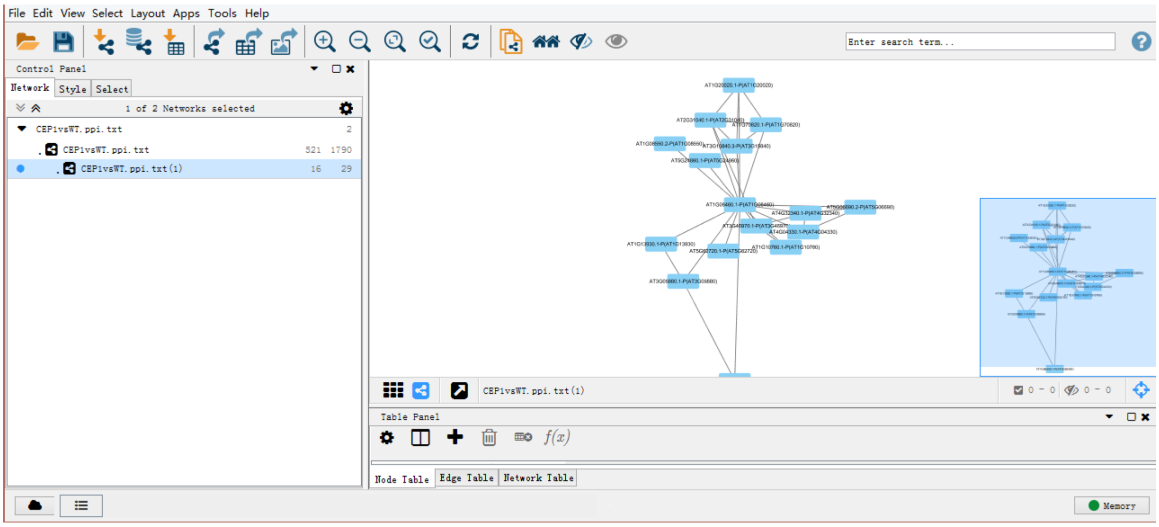

2）网络风格的设置

在 control panel 中选择 Style-->default,设置网络的风格。可以通过拖拽的方式调整节点

的位置。

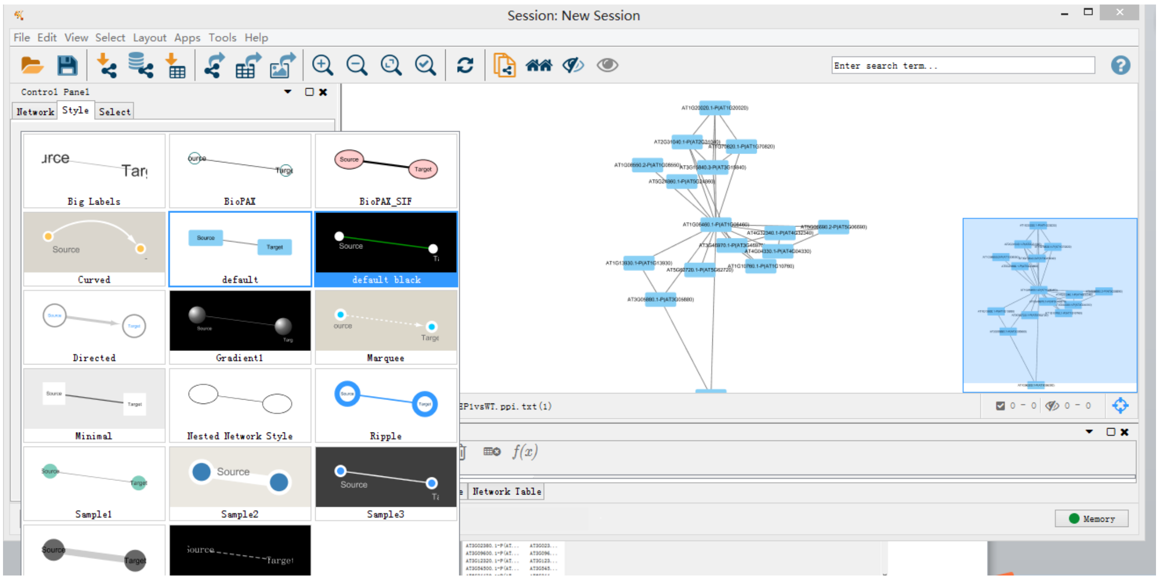

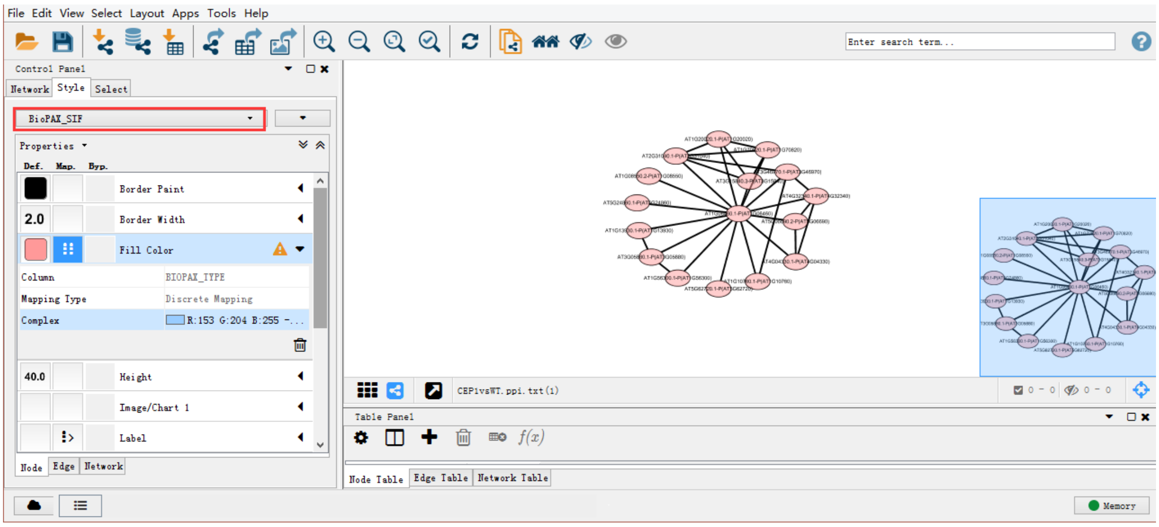

3）节点的个性化设置（节点边框颜色、边框粗细、颜色、标签、透明度等）

以节点的颜色为例：首先选择 control panel 中的 Style,然后选择下方的 Node,点击 Fill Color 进行颜色设置，点击确定。

如需要对单个目标节点进行颜色设定，可以点击 Fill Color-->Column 选择 name；Mapping Type 选择 Discrete Mapping，设置目标节点颜色。

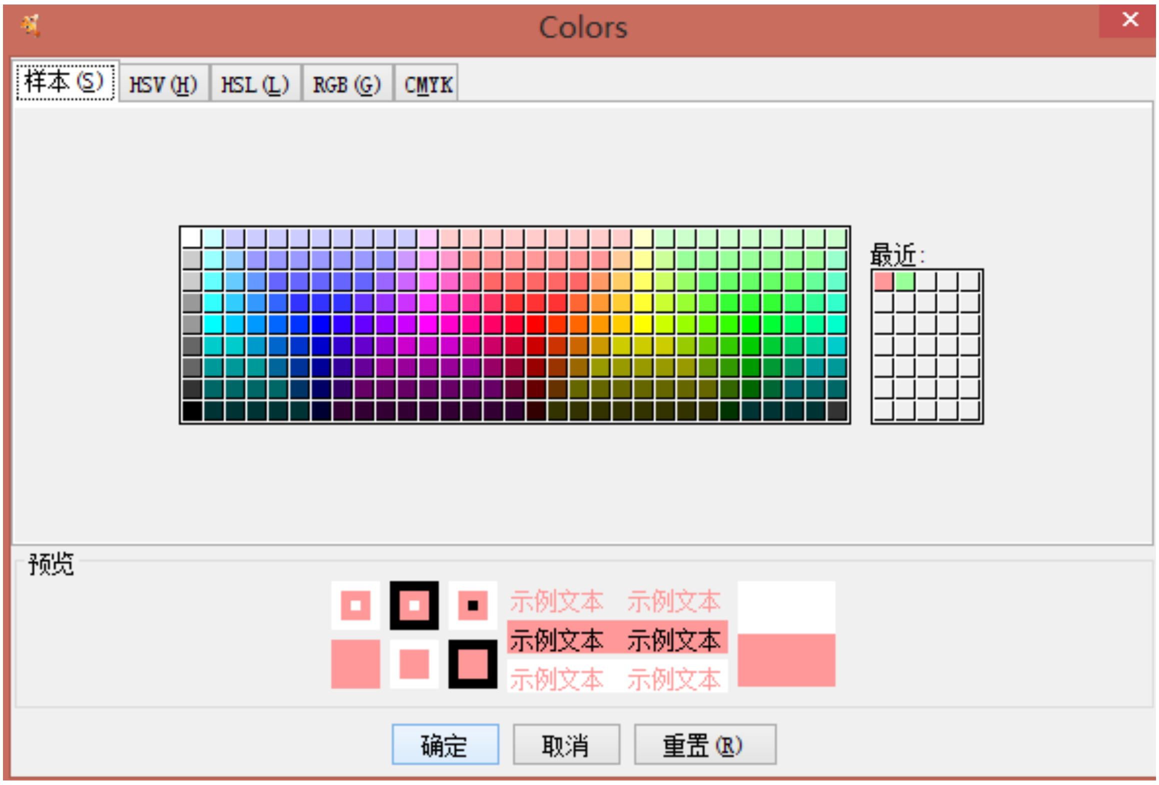

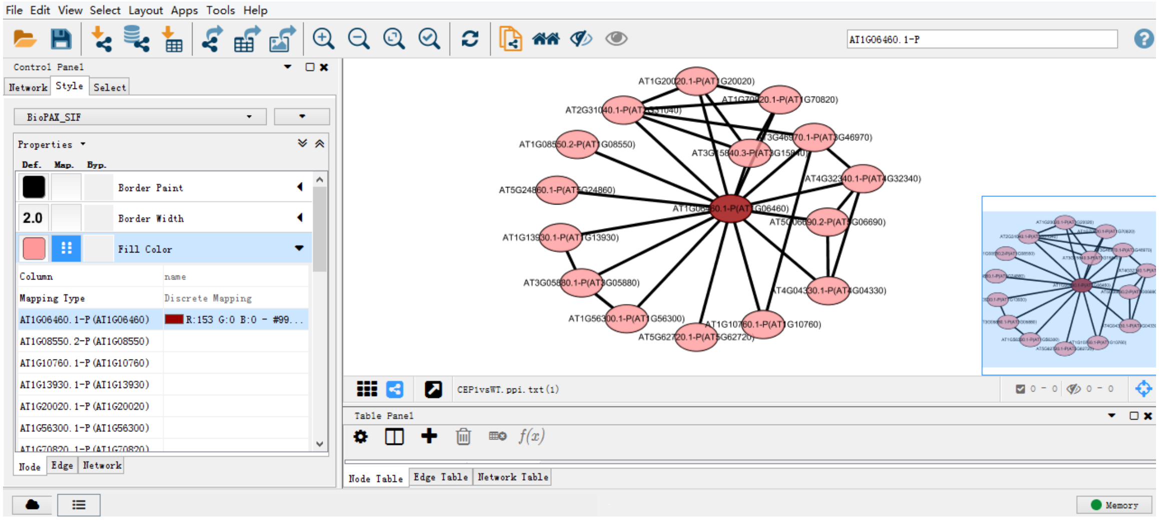

4）边的个性化设置（标签，线形，靶向关系等）

以标签设置为例，将示例中导入的 PPI 数据的蛋白相互作用得分的大小作为边的标签表示出来：选择 control panel 中的 Style，然后选择下方的 Edge—>Lable-->column 选择 interaction; Mapping type 选择 Passthrough Mapping，可以看到边备注了标签。

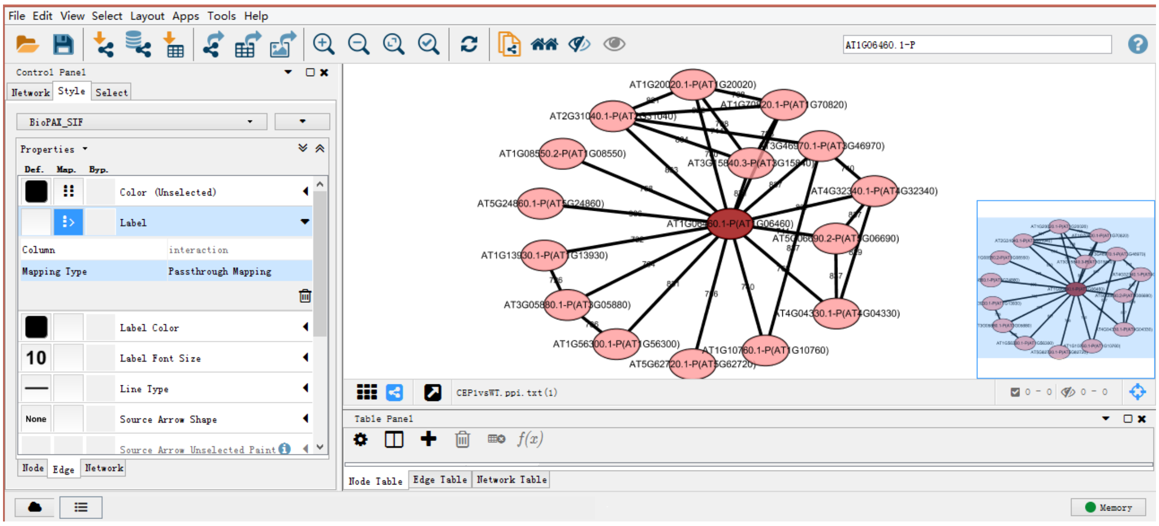

5）网络布局的设置

选择 Layout--> yFiles Layout 选择不同的展示风格。

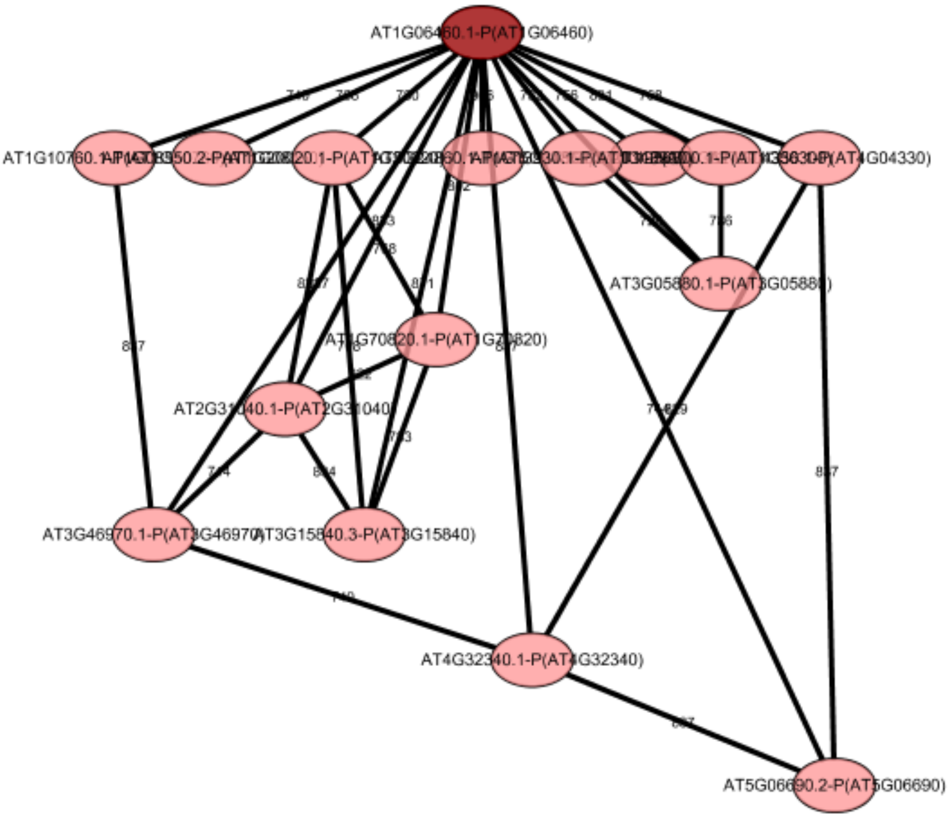

6）网络相关数据的计算

使用 tool 中提供的 Network Analysis 工具可以计算网络相关数据（如节点的 degree

等）信息，进一步完善网络图的丰富度。

选择 Tools-->NetworkAnalysis --> NetworkAnalysis--> NetworkAnalysis-->依据网络是否有方向性选择 directed/undirected--> Visualize Parameters-->

Map Node Size to：选择 degree，代表节点的大小表示节点的 degree 的大小。

Map Node Color to：选择 ClusteringCoefficient，代表节点颜色表示聚类系数。

Map Edge Size to：选择 EdgeBetweenness，代表边的粗细表示边介数。

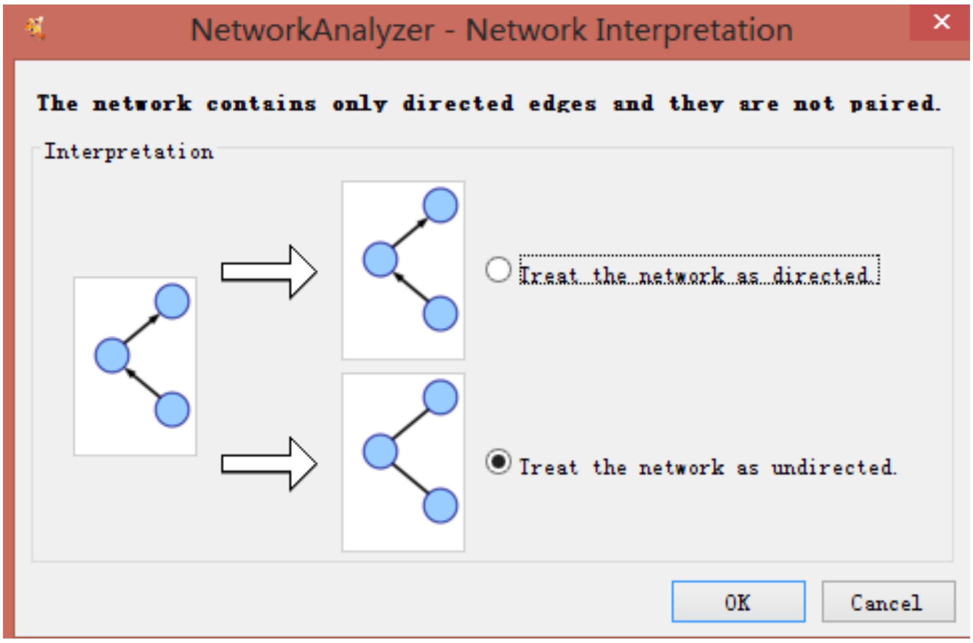

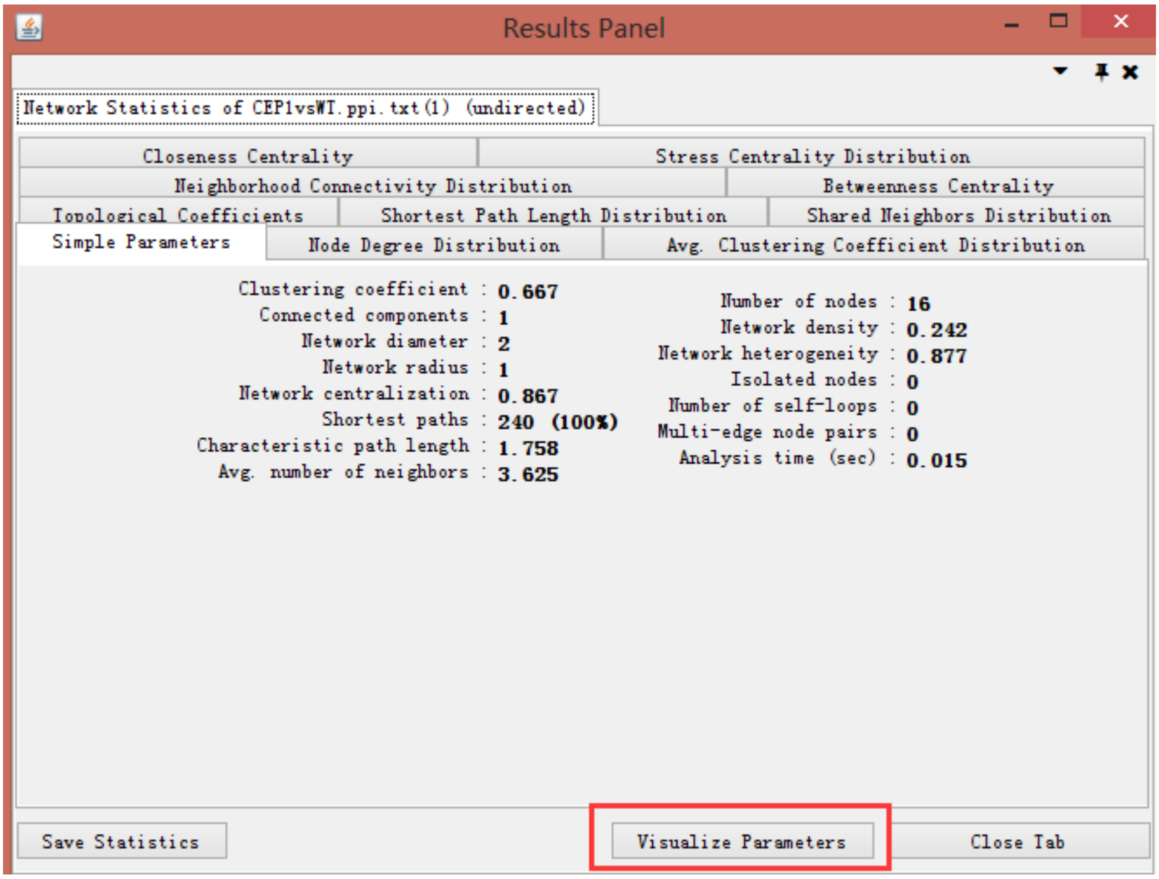
Results Panel
Network Statistics of CEP1vsWT.ppi.txt(1) (undirected)
Closeness Centrality
Stress Centrality Distribution
Neighborhood Connectivity Distribution
Betweenness Centrality
Topological Coefficients
Shortest Path Length Distribution
Shared Neighbors Distribution
Simple Parameters
Node Degree Distribution
Avg. Clustering Coefficient Distribution
Clustering coefficient : 0.667
Connected components : 1
Network diameter : 2
Network radius : 1
Network centralization : 0.867
Shortest paths : 240 (100%)
Characteristic path length : 1.758
Avg. number of neighbors : 3.625
Number of nodes : 16
Network density : 0.242
Network heterogeneity : 0.877
Isolated nodes : 0
Number of self-loops : 0
Multi-edge node pairs : 0
Analysis time (sec) : 0.015
Save Statistics
Visualize Parameters
Close Tab

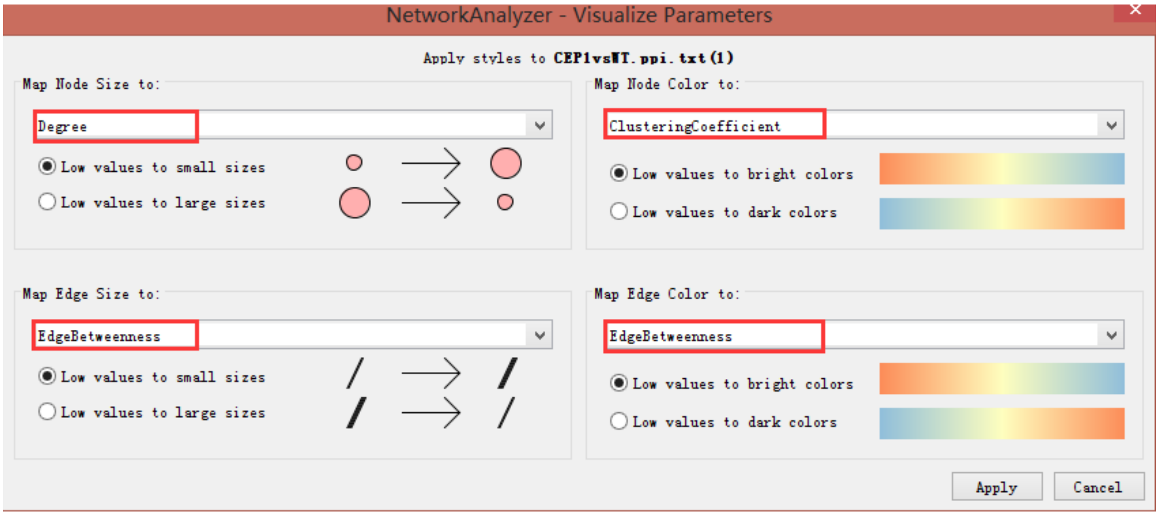
NetworkAnalyzer - Visualize Parameters
Apply styles to CEP1vsWT.ppi.txt(1)
Map Node Size to:
Degree
Low values to small sizes
Low values to large sizes
Map Node Color to:
ClusteringCoefficient
Low values to bright colors
Low values to dark colors
Map Edge Size to:
EdgeBetweenness
Low values to small sizes
Low values to large sizes
Map Edge Color to:
EdgeBetweenness
Low values to bright colors
Low values to dark colors
Apply
Cancel

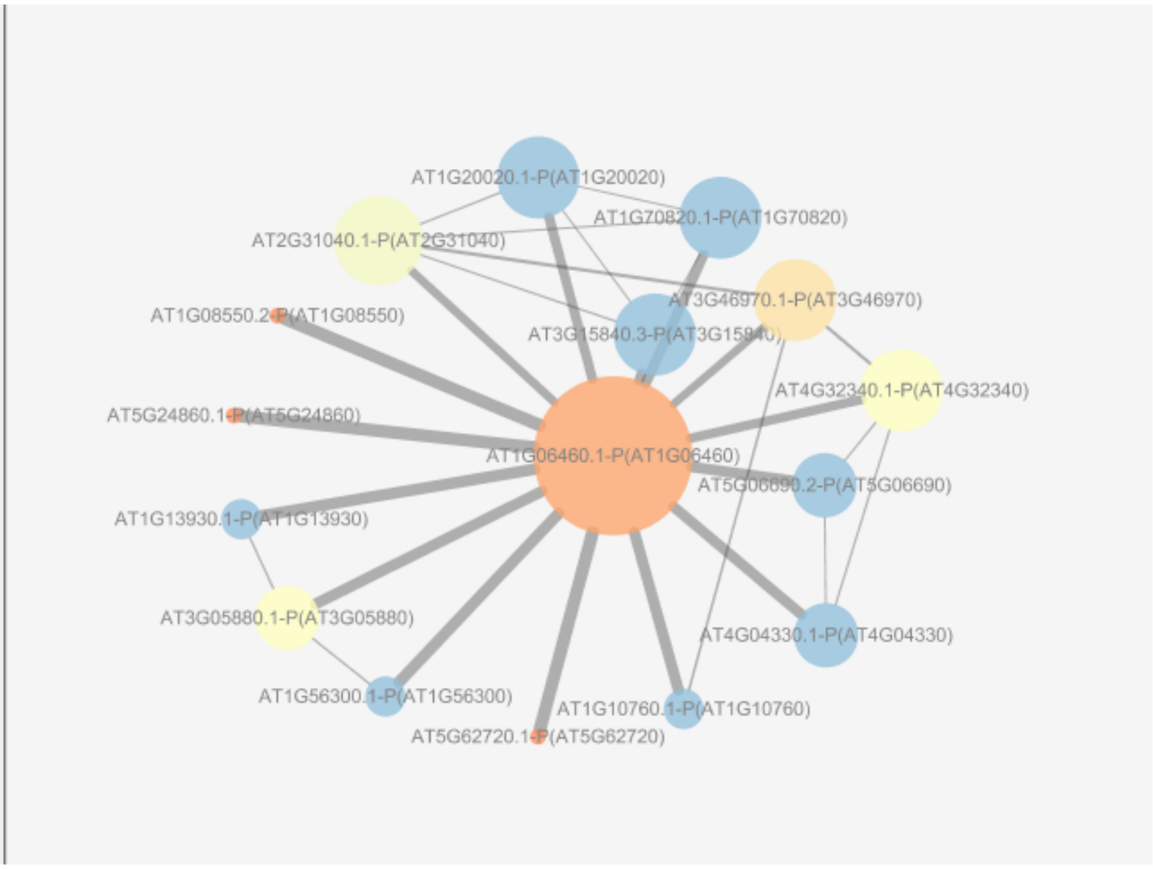

7）网络的保存

Cytoscape 遵循所见即所得的规则，将整张网络图拖拽到网络窗口的中心，然后再保存文件。网络文件保存有多种格式：

格式 1：选择 File-->Save AS 以*.cys 格式保存网络图，该格式文件可以使用 Cytoscape 打开后继续调整网络图。

格式 2：选择 File-->Export-->Export as Image 以 png/PDF 格式保存网络图。

五、 CeRNA 案例演示

1. network 文件的输入：

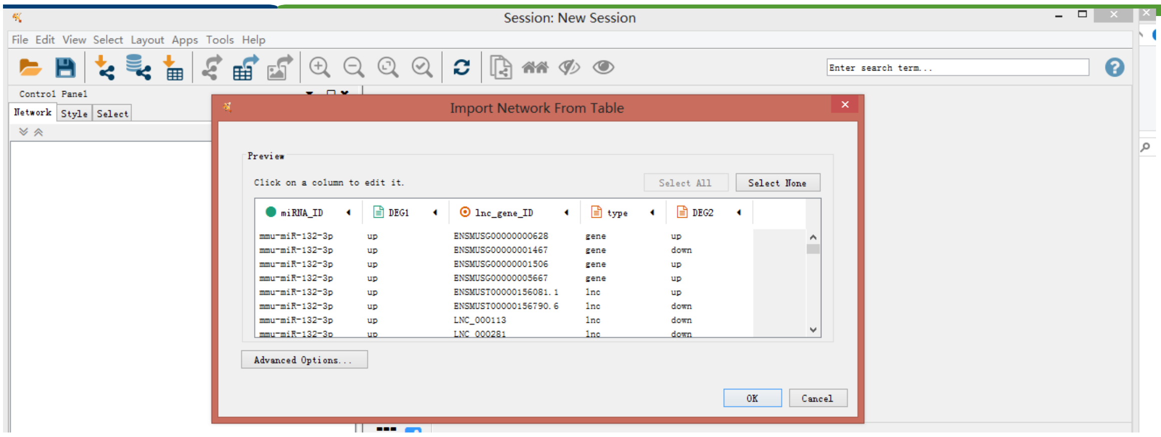

2、注释信息的导入：

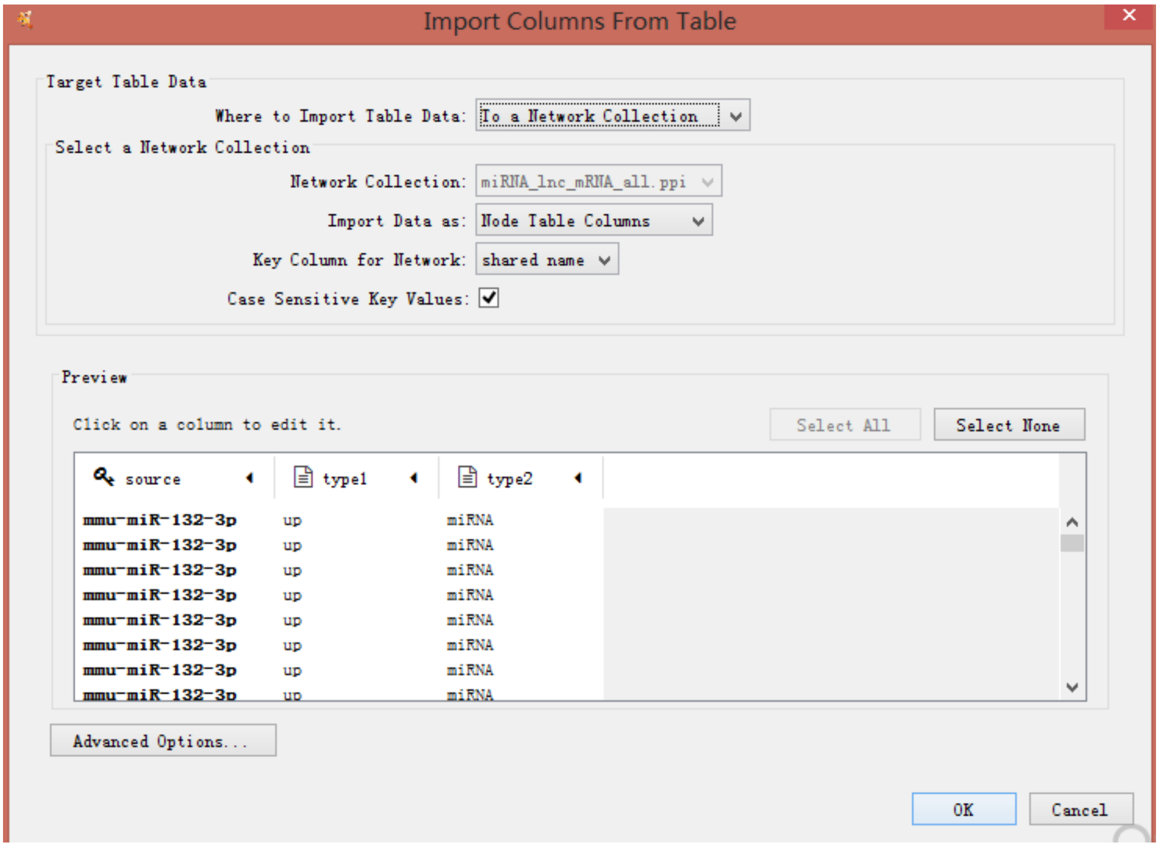

3、对不同类型的 RNA 用边的形状进行分类：

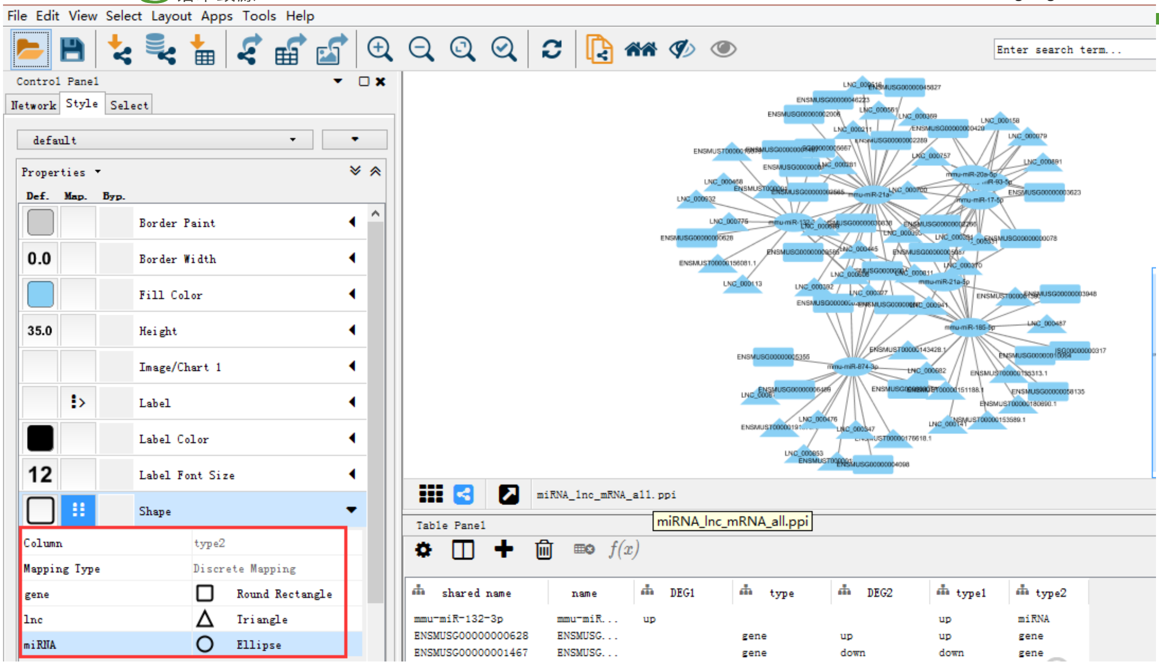

4、对上下调信息用节点颜色区分：

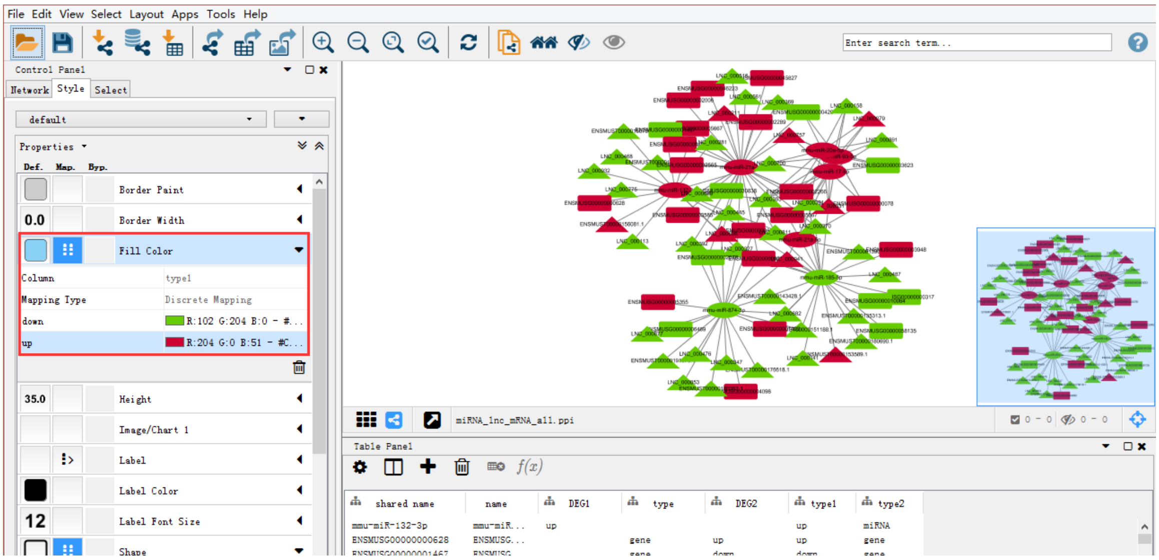

5、节点宽和高的调整：

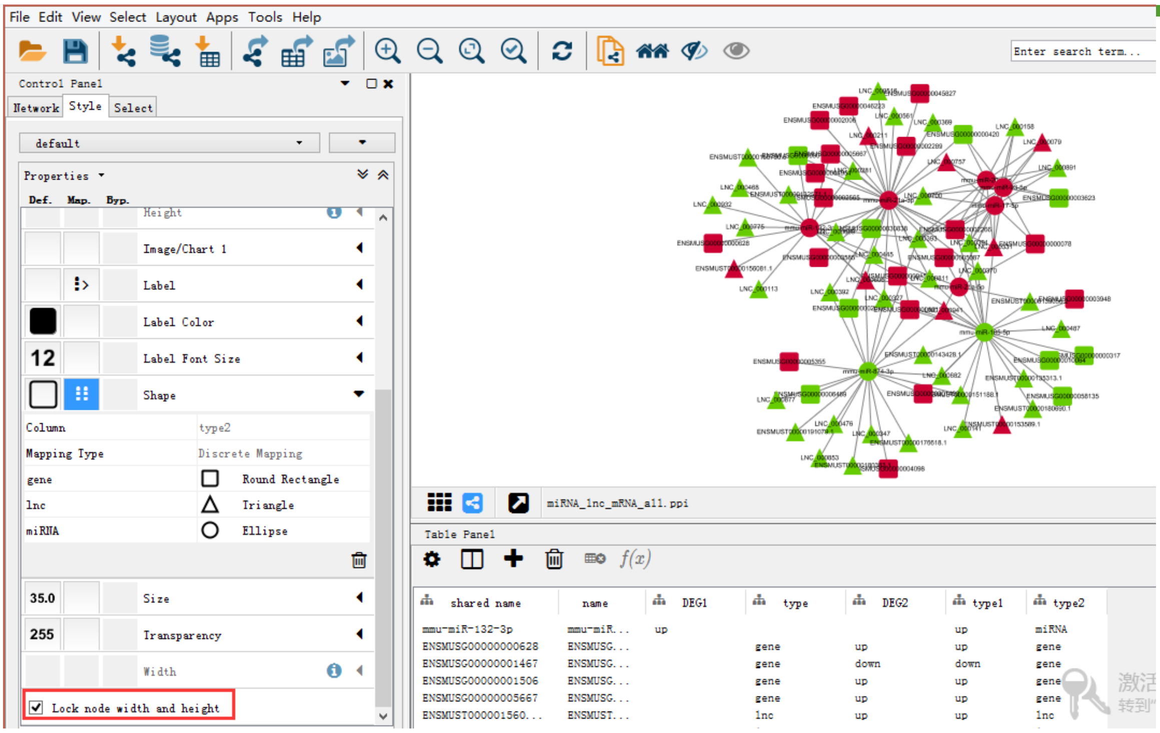

6、结果文件的输出：

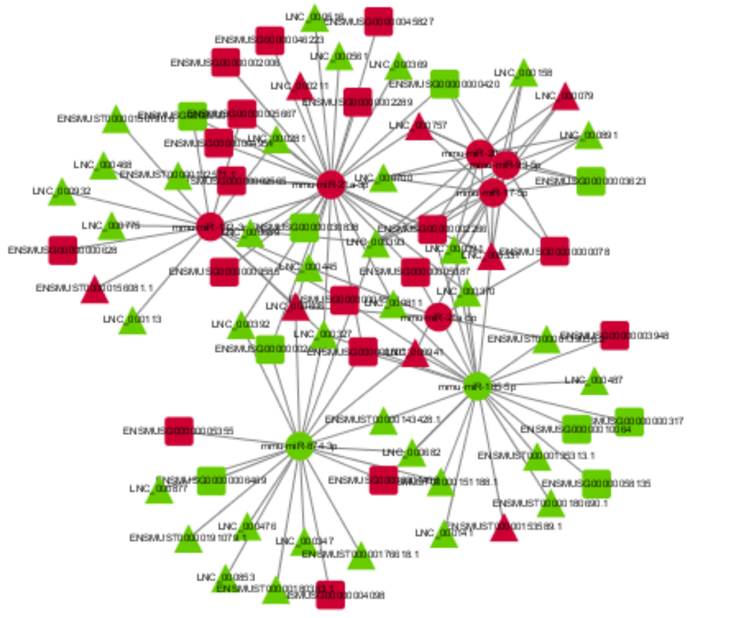

更多请参考：http://wiki.cytoscape.org/Cytoscape_3/UserManual
